# Is deliberate hypotension a safe technique for orthopedic surgery?: a systematic review and meta-analysis of parallel randomized controlled trials

**DOI:** 10.1186/s13018-019-1473-6

**Published:** 2019-12-02

**Authors:** Jia Jiang, Ran Zhou, Bo Li, Fushan Xue

**Affiliations:** 1 Department of Anesthesiology, Beijing Chaoyang Hospital, Capital Medical University, Beijing, 100020 China; 2 Beijing Hospital of Traditional Chinese Medicine, Affiliated with Capital Medical University, Beijing Institute of Traditional Chinese Medicine, Beijing, 100010 China; 3 Department of Anesthesiology, Beijing Friendship Hospital, Capital Medical University, Beijing, 100050 China

**Keywords:** Deliberate hypotension, Orthopedic surgery, Randomized controlled trial, Meta-analysis

## Abstract

**Background:**

Deliberate hypotension has been shown to reduce the intraoperative bleeding and the need for allogeneic blood transfusion, and improve the surgical field, but there is still controversy on its clinical safety. This systematic review was designed to assess the safety and benefits of deliberate hypotension for orthopedic surgery.

**Methods:**

The review met the requirements of the PRISMA guidelines. The Cochrane Central Register of Controlled Trials (CENTRAL), MEDLINE, EMBASE, CINAHL, ISI Web of Science, ScienceDirect, and four Chinese databases (China National Knowledge Infrastructure, Wanfang, vip citation database, and updated version of China Biology Medicine disc from January 1, 2000 to January 1, 2019) were searched. All parallel randomized controlled trials comparing the effects of using deliberate hypotension with not using deliberate hypotension on clinical outcomes of patients undergoing orthopedic surgery were selected. The primary outcome was overall mortality. The secondary outcomes were the intraoperative blood loss, blood transfusion volume, and serious adverse postoperative events.

**Results:**

A total of 30 studies with 36 comparisons (1454 participants) were included in meta-analysis. Two studies with 120 participants reported overall mortality and the result was zero (low-quality evidence). The use of deliberate hypotension reduced the intraoperative blood loss (mean difference, − 376.7; 95% CI − 428.1 to − 325.3; *I*^2^ = 94%; 29 studies, 36 comparisons, and 1398 participants; low-quality evidence) and blood transfusion volume (mean difference, − 242.5; 95% CI − 302.5 to − 182.6; *I*^2^ = 95%; 13 studies, 14 comparisons, and 544 participants; low-quality evidence). Six studies with 286 participants reported the occurrence of serious adverse postoperative events and the result was zero (low-quality evidence). Subgroup analyses according to age groups, controlled mean artery pressure levels, types of orthopedic surgeries, different combinations of other blood conservative method, and hypotensive methods mostly did not explain heterogeneity; significant differences were identified in almost all subgroups.

**Conclusions:**

Based on the available evidence, it is still unclear whether or not deliberate hypotension is a safe technique for orthopedic surgery due to limited studies with very small sample size, though it may decrease the intraoperative blood loss and blood transfusion volume irrespective of age groups, controlled mean artery pressure levels, types of surgeries, hypotensive methods, or different combinations of other blood conservation strategies.

**Trial registration:**

PROSPERO CRD42016045480.

## Background

Orthopedic surgery always involves the manipulation of bone marrow, muscle tissue, and some venous plexus. Due to complex vascularity, bleeding during orthopedic surgery is relatively large and often manifests as diffused bleeding and is not readily controllable by conventional surgical techniques methods, especially when manipulation involves intrabony capillaries [1]. Thus, blood transfusion, in particular the transfusion of red blood cells, is a common practice in orthopedic surgery [2]. In view of potential adverse effects of blood transfusion [3] and an increasing shortage of blood resources, many efforts have been made on the alternatives to transfusion or blood conservation measures to minimize allogeneic blood transfusion, such as acute hypervolemic or normovolemic hemodilution, perioperative blood salvage, use of epoetin alfa to stimulate erythropoiesis, hemostatic agents, deliberate hypotension, and others [4, 5].

Deliberate hypotension refers to any technique that decreases intraoperative blood pressure. Various techniques for deliberate hypotension have been used, including controlling venous return (e.g., changing patient position), and pharmacological interventions (for example, the use of volatile anesthetics, intravenous anesthetics, vasodilators, or β-adrenoceptor antagonists), intrathecal anesthesia, and others. These hypotensive techniques can be used alone, or in combination. The ideal technique should be easy to perform, have a short onset time, an effect that disappears quickly when drug administration is discontinued, a rapid elimination without toxic metabolites, negligible effects on vital organs, and a predictable and dose-dependent effect. It has been shown that the use of deliberate hypotension can shorten the operative time, reduce the risk of tissue edema caused by ligation or electrocautery, and improve myocardial performance by reducing cardiac preload and afterload [4, 6]. A recent retrospective cohort study indicates that deliberate hypotension has a potential ability to minimize length of hospital stay for patients undergoing orthognathic surgery [7]. In 2007, moreover, a meta-analysis provides evidence to support for the use of deliberate hypotension in orthopedic surgery [8]. However, deliberate hypotension has a potential risk of multiple complications. Even within “a safe range of hypotension,” brain damage, stroke, and death may still occur [9]. It is reported in the early 1950s that mortality associated with deliberate hypotension is about 0.22% to 0.34%, and nonfatal complications mainly referred to cerebral, coronary, and renal circulations occur 908 times (about 2.6% to 3.3%) [10]. In the early 1960s, a mortality of 0.10% is reported in 9107 patients with deliberate hypotension [11]. Thus, clinical safety of deliberate hypotension has always been a major concern of clinicians, especially for patients with known hypertension, elderly patients, and those requiring special positions during surgery (e.g., beach-chair position and reverse Trendelenburg position). Nevertheless, no systematic review and meta-analysis on the safety of this technique has been conducted until now. Moreover, the benefits of deliberate hypotension for orthopedic surgery have not been updated since 2007. Thus, this systematic review was performed to assess the safety and benefits of deliberate hypotension for orthopedic surgery.

## Methods

### Eligibility criteria

This systematic review and meta-analysis was conducted following the recommendations of the Cochrane Handbook for Systematic Reviews and Interventions and reported according to the PRISMA statement (www.prisma-statement.org) [12]. The protocol had been registered on the PROSPERO (http://www.crd.york.ac.uk/PROSPERO CRD42016045480).

All parallel randomized controlled trials (RCTs) comparing the effects of using deliberate hypotension with not using deliberate hypotension for orthopedic surgery on any primary and secondary outcomes were included, irrespective of language or publication status. Observational studies, randomized cross-over trials, prospective cohort studies, and quasi-randomized studies were excluded. All orthopedic surgical participants irrespective of ages, sexes, or anesthetic methods used were included. Spinal surgery performed by neurosurgeons was also included if deliberate hypotension was used during surgery. Patients scheduled for orthognathic surgery or those with a history of neurologic or psychiatric dysfunction, uncontrolled hypertension, ischemic heart diseases, stroke, renal or hepatic dysfunction, severe peripheral vascular diseases, uncorrected hypovolemia, and anemia (hemoglobin level ≤ 110 g/dL) were excluded. The intervention group used deliberate hypotension by any method. For the control group, blood pressure was not specifically controlled. Studies combining deliberate hypotension with any method of hemodilution (hypervolemic or normovolemic), cell salvage, tourniquet, or other pharmacological interventions (use of hemostatic agents such as hemocoagulase, tranexamic acid, etc.) to reduce blood loss were also included if they were applied equally to groups. The primary outcome was overall mortality. The secondary outcomes were intraoperative blood loss, blood transfusion volume, and serious adverse postoperative events. The definition of outcomes was summarized in Additional file 1: Table S1.

### Search strategy

The current issue of the Cochrane Central Register of Controlled Trials (CENTRAL), MEDLINE (Ovid SP), EMBASE (Ovid SP), CINAHL (via EBSCOhost), ISI Web of Science, ScienceDirect (via Elsvier), and four Chinese databases: China National Knowledge Infrastructure (CNKI), Wanfang, vip citation database (VIP), and SinoMed (updated version of China Biology Medicine disc) from January 1, 2000 to January 1, 2019, with no date/time, language, and document type limitations, were searched. Subject search terms with the Cochrane highly sensitive strategies for identifying RCTs described in Section 6.4 of the Cochrane Handbook for Systematic Reviews of Interventions [13] were used to search MEDLINE. The MEDLINE search strategy was applied to search other electronic databases. Keywords were collected through experts’ opinion, literature review, controlled vocabulary (medical subject headings = MeSH and Excerpta Medica Tree = EMTREE), and reviewing the primary search results. The BIOSIS databases (http://www.biosis.org/), SIGLE database (opensigle.inist.fr), and HMIC database (www.ovid.com/site/catalog/ DataBase/99.jsp?top=2&mid=3&bottom=7&subsection=10) for conference proceedings and grey literature were also searched. Websites of www.clinicaltrials.gov and www.controlled-trials.com/ were searched to identify unpublished trials from January 1, 2000 to January 1, 2019. All search strategies developed by assistance of a medical information specialist were reported in Additional file 2. For literature without full text, we planned to email the study author. The reference lists were screened of all eligible trials and reviews identified for further references to additional trials.

### Study selection

All search results were imported into the Endnote Software by two study authors (JJ and ZR) and duplicate records were removed. If uncertainties remained, the corresponding study author was contacted. Then the title and abstracts were independently screened (JJ and ZR). Any obviously irrelevant studies were removed. After retrieving the full texts of any potentially relevant studies, their eligibility was carefully determined. Any disagreement between the two review authors was resolved by discussion with other authors (XFS and LB).

### Data extraction and management

Data was independently extracted by two review authors (JJ and ZR) and entered in our prespecified data collection form (Additional file 2: Table S2). For the continuous data, mean, standard deviation (SD), and sample size were extracted; for studies that only reported median and interquartile range (IQR), median was considered as similar as mean and IQR was approximately 1.35 SD [13]. For the dichotomous variables, the number of events occurred and the sample size were extracted. For rare events that might re-occur to a person or several rare events concurring in one person during the study follow-up period (Poisson data), the total number of events in each group and the total number of person-time at risk in each group were extracted; rates related the counts to the amount of time during which they could have happened [13]. Since a fixed time-point (28 days) to follow was set, the time-to-event outcome (mortality) was treated as dichotomous data. For the information that was unable to extract from the available report, the original study authors were contacted. Any disagreements in data extraction were resolved by discussion between two review authors, and if necessary, with a third review author (XFS).

### Assessment of risk of bias in included studies

The risk of bias for each eligible study was assessed by using the “Risk of bias” assessment tool and a “Risk of bias” summary figure was generated by using Review Manager 5.3.5 software. Any disagreement on this assessment was resolved by discussion with a third review author (XFS). For assessment of the risk of bias within and across the included studies, the approach provided in the Cochrane Handbook for Systematic Reviews of Interventions was followed to rate them as unclear, low, or high risk study [13]. The criteria of the GRADE system was used to assess the quality of body of evidences associated with all outcomes [14]. Then a “Grade evidence profile” table was developed by using the GRADE software (www.guidelinedevelopment.org) to rate these outcomes as high, moderate, low, or very low quality. The quality of evidence was downgraded by one or two levels when serious or very serious deficiencies were considered in these criteria. Reporting bias was qualitatively assessed by using funnel plot if the result of the primary outcome was from at least ten trials [15].

### Measures of treatment effect

The risk ratio (RR) and 95% confidence interval (CI) for dichotomous data were used. The mean difference (MD) and 95% CI for continuous data were used when the outcomes in all included studies were made on the same scale. For the rare events that might re-occur to a person during the study follow-up period, such counts were treated as rate data. In addition, trial sequential analysis (TSA) was planned to calculate the required information size for primary outcome and one of secondary outcomes (occurrence of serious adverse postoperative events). The calculation was based on the rate of our control group and the statistics with α and β error of 0.05 and 0.20 (two-sided test) and RR reduction of 20%, then the calculated sample size was multiplied by the heterogeneity in our result [16]. *P* < 0.05 was considered as statistically significant.

For the studies with more than two intervention groups, such as experimental groups with different methods to induce hypotension, or combined with other methods to modulate blood loss (e.g., hemodilution), or one experimental group with two control groups, the “shared” group with similar sample size was split and two or more comparisons were created.

The study author of original report was contacted for important missing statistics. If these data still could not be obtained, the available data was used. If no usable data could be extracted from an eligible study, potential implications of missing data were discussed instead of excluding the study from this review. For the participants’ missing due to drop-out, if “missing at random,” analysis was performed based on the available data; if not, an available case analysis was performed or if necessary, an intention-to-treat (ITT) analysis was planned. If the study did not mention withdrawals, no drop-outs were assumed.

### Data synthesis

Review Manager 5.3.5 software was used to perform the pooled analysis for the outcomes from more than one study. A chi^2^ test with the *I*^2^ statistic (with statistical significance set at the level of two-tailed 0.10) was used to describe the percentage of the total variance across studies from heterogeneity rather than from chance. When *I*^2^ is less than 40%, namely there was no statistical heterogeneity among studies, a fixed-effect model was used; otherwise, a random-effect model was used. In case of evidence of significant heterogeneity, results of both fixed-effect and random-effect models were compared to evaluate if the small study effect had an influence on the treatment effect estimate. If an outcome originated from data of only one study, the estimate of effect was calculated from this single study. For the results that could not be analyzed via meta-analysis, only a qualitative systematic review was performed.

### Subgroup analysis and investigation of heterogeneity

Heterogeneity (clinical and methodological) was considered before performing pooled analysis. Subgroup analyses were performed in the presence of statistical heterogeneity (*I*^2^ ≥ 40%) or an indication of clinical heterogeneity [13]; the following subgroups were considered: (a) age groups: younger than 16 (children), 17 to 65 (adults), older than 65 years of age (elderly patients); (b) controlled mean artery pressure (MAP) levels: ≥ 60 mmHg, 55 to 60 mmHg, and < 55 mmHg; meta-regression was planned to assess the relationship between controlled MAP levels and the primary outcome if no less than ten studies reported the primary outcome were included in the review; (c) types of orthopedic surgeries; (d) different combinations of other blood conservative method; and (e) hypotensive methods used.

### Sensitivity analysis

In order to determine the robustness of our meta-analysis, sensitivity analyses by sequentially removing each high risk study was conducted and the remaining data set for the primary outcome was reanalyzed. Additionally, a previous study suggested that the probability of positive results reported in studies of certain languages, such as Chinese, were significantly higher than other languages [17]. Therefore, a “special” sensitivity analysis was planned by excluding Chinese studies to confirm if the Chinese articles affect the results of pooled analysis for the primary outcome.

## Results

### Description of studies

The results were presented using the PRISMA statement method [18].

### Results of the search

**Fig. 1:**
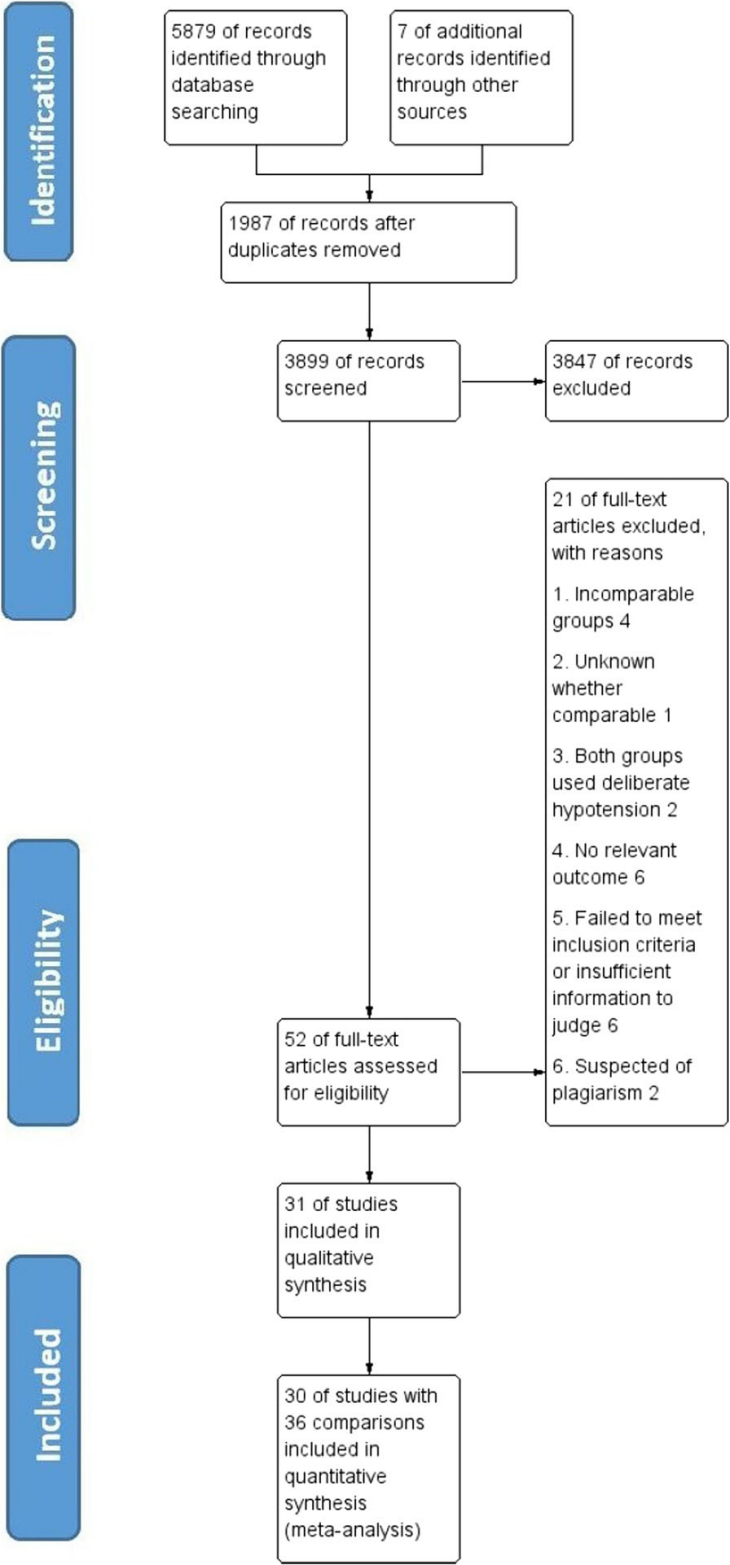
The PRISMA flow chart of included and excluded studies

Using Search strategy, a total of 5886 records were identified. They were de-duplicated (1987 removed) in EndNote X5 and then sent to two researchers (JJ and ZR) for screening. Further, 3847 were excluded during screening as they were irrelevant to our research question or non-RCT. Fifty-two studies were selected for full text assessment using inclusion and exclusion criteria. Four studies [19–22] were further removed as other blood conservative methods were applied unequally to groups; one [23] because of insufficient information to judge whether the two groups were comparable; two [24, 25] because both groups used deliberate hypotension; six [26–31] because of no relevant outcomes; six [32–37] because of failing to meet inclusion criteria or insufficient information to make a judgment; and two [38, 39] because of plagiarism suspected. Thus, 31 studies with 1504 participants) were selected in qualitative synthesis [40–70]; among them, seven studies had two comparisons [45, 53, 59, 61, 64, 65, 70] and one study had no data available (the author’s contact information was unavailable) [63]. Finally, 30 studies with 36 comparisons (1454 participants) were included in quantitative synthesis (data extraction for meta-analysis). Process of selection of studies has been shown in PRISMA flow diagram [12] (Fig. 1).

Twenty-four comparisons in 20 studies compared the deliberate hypotension versus control (no deliberate hypotension) [42–44, 49, 51–53, 55–61, 63–65, 68–70]; four comparisons in three studies compared deliberate hypotension combined with acute normovolemic hemodilution versus acute normovolemic hemodilution [40, 45, 47]; seven comparisons in seven studies compared deliberate hypotension combined with acute hypervolemic hemodilution versus acute hypervolemic hemodilution [41, 48, 53, 62, 64, 66, 67]; two studies compared deliberate hypotension combined with acute hypervolemic hemodilution and cell salvage versus acute hypervolemic hemodilution combined with cell salvage [46, 50]; one study compared deliberate hypotension combined with cell salvage versus cell salvage [54]. Most of the included studies enrolled patients aged 17 to 65 years; four included elderly patients [43, 54–56], and seven studies included both adult and elderly patients [44, 46, 49, 50, 52, 58, 62]; four studies did not clearly describe this issue [60, 61, 63, 68]. The types of surgeries included spine surgery, total hip arthroplasty, pelvic surgery, or femoral fracture surgery. The types of surgeries were not clarified in four studies [41, 42, 45, 53]. Eight methods of deliberate hypotension were investigated: remifentanil [40–42, 45, 59, 66], nitrates [43, 45, 48, 50, 51, 53, 55, 59, 61–64], sevoflurane [46, 54], esmolol [47], milrinone [49], epidural anesthesia [58], nicardipine [60, 68, 70], and a combination of two or three hypotensive drugs [44, 52, 56, 57, 61, 65, 67, 69, 70]. The controlled MAP level in 14 studies was ≥ 60 mmHg [40, 43, 50, 54, 56, 57, 60–62, 64, 66, 68–70]; seven studies only limited the percentage of hypotension [44, 45, 51–53, 63, 67]; one study controlled the MAP between 45 and 50 mmHg; other studies covered two or more hypotensive level groups. Fourteen studies did not provide the transfusion trigger points [41, 43, 44, 46, 50, 52, 55–57, 60, 63, 65, 68, 69]. Thirteen studies described the method of measuring the volume of blood loss; most of which was defined as “blood collected in the suction bottles and the increased weight of gauzes” [41, 42, 46, 48, 49, 51, 53, 57, 58, 60, 63, 64, 67]. The characteristics of the included studies was summarized in Table 1.

**Table 1:** Characteristics of included studies

Study ID	Locations	Sample sizes	Intervention groups	Comparison groups	Age range (years)	MAP levels (mmHg, DH group)	MAP levels (mmHg, C group)	Definitions of intraoperative blood loss	Types of surgeries	Anesthesia	Overall mortality	Occurrence of serious adverse events
An 2015 [40]	China	60	Remifentanil	DH + ANH vs. ANH	37–64	60–70	70–80	Not stated	Complicated orthopedic	GA	Y (24h post-op)	Y (24h post-op)
Cheng 2003 [41]	China	30	Remifentanil	DH+AHH vs. AHH	19–65	55–65	89 ± 9.1	Increased weight of gauzes	Orthopedic	GA	N (24h post-op)	N (24h post-op)
Chi 2005 [42]	China	29	Remifentanil	DH vs. C	22–61	55–65; 65 ± 7	90 ± 10	Blood collected in suction bottles and increased weight of gauzes	Orthopedic (spine or joint)	GA	N (> 1 h post-op)	N (> 1 h post-op)
Diao 2006 [43]	China	40	Nitrates	DH vs. C	71–79	70% of baseline; no less than 60	Not stated	Not stated	THA	GA	N (4 days post-op)	N ( days post-op)
Dong 2012 [44]	China	54	Drug combination	DH vs. C	44–78	70% of baseline	86 ± 8	Not stated	THA	GA	N (4 days post-op)	N (4 days post-op)
Fang 2011 [45]	China	37	Remifentanil	DH + ANH vs. ANH	38–65	70% of baseline	ANH: 87 ± 4; C: 85 ± 6	Not stated	Orthopedic	GA	N	N
		38	Nitrates	DH + ANH vs. ANH								
Fukusaki 2008 [46]	Japan	30	Sevoflurane	DH + AHH+ A vs. AHH+ A	51–70	55	95	Weighing swabs and measuring blood collected from wound drainage.	THA	GA	N (7 days post-op)	Y (7 days post-op)
Han 2006 [47]	Korea	56	Esmolol	DH + ANH vs. ANH	< 65	55–65 (57.3 ± 4.4)	85.1 ± 5.7	Not stated	Posterior lumbar fusion	GA	N (7 days post-op)	Y (1 week post-op)
Hu 2005 [48]	China	20	Nitrates	DH + AHH vs. AHH	38–65	55–65	70–100	Increased weight of gauzes.	THA	SA + EA	N	N
Hwang 2013 [49]	Korea	40	Milrinone	DH vs. C	60–70	≤ 60	Not stated	Blood in suction bottle and weighing the wet gauzes	Posterior lumbar fusion	GA	N	N
Jin 2008 [50]	China	60	Nitrates	DH + AHH + A vs. AHH + A	48–66	65 ± 5	86.7 ± 10.3	Not stated	THA	GA	N (unclear)	Y (unclear)
Kazemi 2006 [51]	Iran	60	Nitrates	DH vs. C	20–60	85% of baseline	94% of baseline (85–95)	Blood in suction container and degree of wetness of used sponges were estimated	Open fixation of femur fracture	GA	Y (unclear, during hospital stay)	Y (unclear, during hospital stay)
Li 2012 [52]	China	50	Drug combination	DH vs. C	51–86	70% of baseline	Not stated	Not stated	THA	EA+SA	N	N
Liang 2004 [53]	China	24	Nitrates	DH + AHH vs. AHH	41–63	70% of baselineDH + AHH: 61 ± 6; DH: 63 ± 4	AHH: 89 ± 8; C: 95 ± 12	Blood collected in suction bottles and estimated blood in gauzes	Orthopedic	GA	N (24 h post-op)	N (24 h post-op)
		24	Nitrates	DH vs. C								
Liu 2009 [55]	China	60	Nitrates	DH vs. C	65–82	Decreased by 30% of baseline, and not less than 55	86 ± 16	Not stated	THA	GA	N (4 days post-op)	N (4 days post-op)
Liu 2011 [56]	China	40	Drug combination	DH vs. C	65–82	70% baseline and no less than 60	88 ± 5	Not stated	THA	GA	N (4 days post-op)	N (4 days post-op)
Liu 2014 [54]	China	60	Sevoflurane	DH + A vs. A	65–83	65–75	86.2 ± 8.6	Not stated	Unilateral THA	GA	N (48 h post-op)	N (48 h post-op)
Luo 2011 [57]	China	60	Drug combination	DH vs. C	30–62	55.7 ± 6.9	85.3 ± 10.4	Blood collected in suction bottles, blood loss of gauzes and salt water towel.	Spine surgery	GA	N	N
Niemi 2000 [58]	Finland	30	Epidural anesthesia	DH vs. C	Not stated	50–60	Not stated	Contents of suction bottles and increase in weight of surgical swabs	Unilateral THA	EA in DH group and SA in C group	N (next morning of surgery)	N (next morning of surgery)
Wang 2012 [59]	China	23	Remifentanil	DH vs. C	50–65	70% of baseline (no less than 55):	79 ± 12	Not stated	THA	GA	N	N
		22	Nitrates	DH vs. C								
Wu 2000 [60]	China	20	Nicadipine	DH vs. C	Not stated	60–65	95 ± 4.6	Blood collected in suction bottles and the increased weight of gauzes.	Intervertebral disc pick out and vertebra planted bone	GA	N (unclear)	Y (unclear)
Xiao 2005 [61]	China	37	Nitrates	DH vs. C	Not stated	60–65	Not stated	Not stated	Internal fixation of vertebral or pelvic fracture; spinal tumor resection	GA	N	N
		38	drug combination	DH vs. C								
Xiong 2015 [62]	China	40	Nitrates	DH + AHH vs. AHH	50–73	65 ± 5	Not stated	Not stated	THA	GA	N	N
Yang 2002 [63]	China	50	Nitrates	DH vs. C	Not stated	60–70% of baseline	Not stated	Blood collected in suction bottles and the increased weight of gauzes.	Cervical posterior decompression	GA	N	N
Yuan 2015 [64]	China	40	Nitrates	DH + AHH vs. AHH	48–62	65 ± 5	Not stated	Blood collected in suction bottles and increased weight of gauzes.	Internal fixation of thoracolumbar spine	GA	N	N
		40	Nitrates	DH vs. C								
Zhai 2011 [65]	China	30	Drug combination	DH vs. C	18–59	55–65	Not stated	Not stated	Posterior lumbar decompression and bone grafting	GA	N (5 days post-op)	N (5 days post-op)
		30				45–50						
Zhang 2009 [66]	China	20	Remifentanil	DH + AHH vs. AHH	22–54	68 ± 5	AHH: 83 ± 5; C: 86 ± 7	Not stated	Fixation of vertebral pedicle	GA	N	N
Zhu 2006 [68]	China	40	Nicadipine	DH vs. C	Not stated	60–65	Not stated	Not stated	THA; internal fixation of femoral fracture	GA + EA	N (2 days post-op)	N (2 days post-op)
Zhu 2007 [67]	China	24	Drug combination	DH + AHH vs. AHH	35–65	75% of baseline	AHH: 70–90; C: 70–100	Blood collected in suction bottles and increased weight of gauzes	Spinal surgery	GA	N (1 day post-op)	N (1 day post-op)
Zhu 2009 [69]	China	60	Drug combination	DH vs. C	25–58	60–65	Not stated	Not stated	THA; internal fixation of femoral fracture	GA	N (2 days post-op)	N (2 days post-op)
Zhu 2013 [70]	China	45	Nicadipine	DH vs. C	30–60	60–65	Not stated	Not stated	THA, internal fixation of femoral fracture	GA	N (2 day post-op)	N (2 day post-op)
		45	Drug combination									

*DH* deliberate hypotension, *C* control, *ANH*: acute normovolemic hemodilution, *AHH* acute hypervolemic hemodilution, *A* allogeneic blood transfusion with cell salvage, *MAP* mean artery pressure, *GA* general anesthesia, *EA* epidural anesthesia, *SP* spinal anesthesia, *THA* total hip arthroplasty, *post*-*op* postoperatively, *Y* reported, *N* (time point) no drop-out during specified postoperative observational period, *N* not reported

### Risk of bias in included studies

**Fig. 2:**
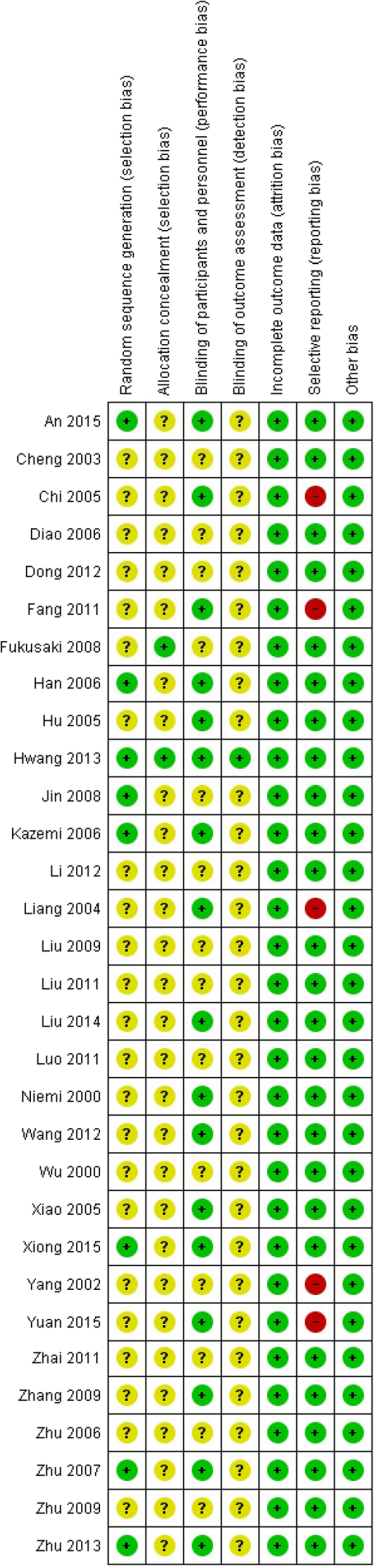
Risk of bias summary: judgments about each risk of bias item for each included study. Quality evaluation of 31 included studies. +, low risk; −, high risk; ?, unknown

Risk of bias for each study was summarized in Fig. 2 and Additional file 2: Table S3. All included studies except one [49] were classified as unclear or high risk study. The evidence for most of the outcomes was graded as low-quality for imprecision due to very small sample size or for inconsistency due to high level of heterogeneity (Additional file 2: Table S4). The funnel plot indicating publication bias, pre-specified sensitivity analysis, meta-regression, and trial sequential analysis for primary outcome could not be done because of limited studies and zero events.

### Effects of interventions

#### Primary outcome—overall mortality

Two studies including 120 participants reported this outcome, one was followed up for 24 h after surgery [40] and the other was probably followed up during hospital stay [51]; the overall mortality was zero. Seventeen studies including 800 participants although did not explicitly observe the mortality, the occurrence of no death during the observation period could be justified according to pre- and postoperative data of the effects on vital organs, which implied no drop-outs occurred [41, 43, 44, 46, 47, 50, 53–56, 58, 60, 65, 67–70].

#### Secondary outcomes

##### Intraoperative blood loss

**Fig. 3:**
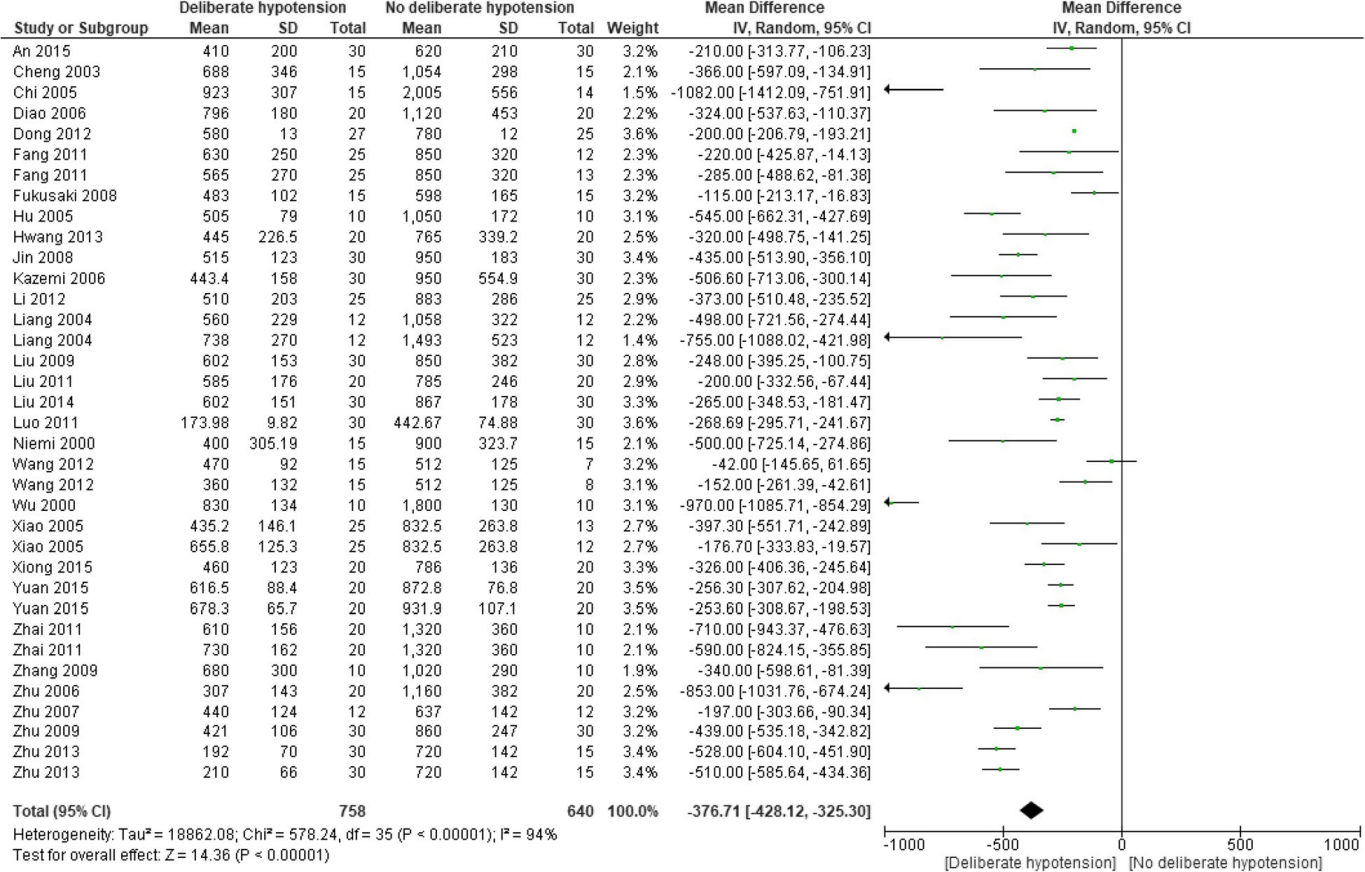
Forest plot for comparison of intraoperative blood loss between deliberate hypotension and no deliberate hypotension. *IV* inverse variance

Thirty studies with 37 comparisons including 1448 participants reported intraoperative blood loss. As one study did not report SD or *P* value, the data could not be used [63]. Finally, 29 studies with 36 comparisons including 1398 participants were included in pooled analysis [40–46, 48–62, 64–70]. The intraoperative blood loss was reduced by 376.7 ml in the intervention group compared with control group (95% CI − 428.1 to − 325.3; *P* < 0.00001; *I*^2^ = 94%; Fig. 3). Subgroup analysis according to age groups, controlled MAP levels, types of surgeries, or different combination of other blood conservative methods or hypotensive methods did not explain heterogeneity, except for “elderly” group and deliberate hypotension combined with acute normovolemic hemodilution versus acute normovolemic hemodilution group (*I*^2^ = 0); and significant differences were identified in all subgroups (*P* <0.01) except one (deliberate hypotension combined with acute hypervolemic hemodilution and cell salvage versus acute hypervolemic hemodilution combined with cell salvage group); the reason for not reaching significant difference for this subgroup might be the not-overlapped CI of two included studies in this group; pooling them together might make no sense (Additional file 2: Figures S1-S5).

##### Blood transfusion volume

**Fig. 4:**
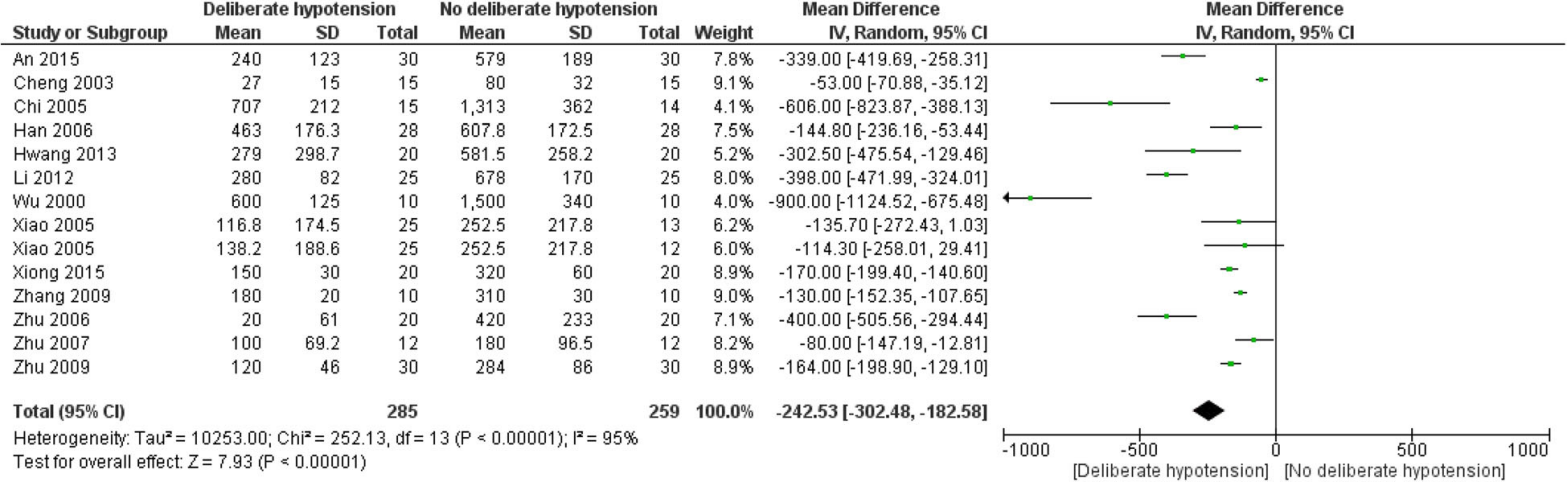
Forest plot for comparison of blood transfusion volumes between deliberate hypotension and no deliberate hypotension. *IV* inverse variance

One study with two comparisons applied acute normovolemic hemodilution, and all the bloods transfused in three groups during surgery were the autologous bloods collected before surgery; no significant differences in the blood transfusion volumes were identified among groups [45]. Five studies reported the number [51, 56, 59, 64] or proportion [53] of patients needing blood transfusion; all of them reported a higher number of patients needing blood transfusion in the control group than in the intervention group, and the data could not be included in meta-analysis. Eighteen studies with 20 comparisons including 754 participants reported blood transfusion volume. The intervention groups in five studies did not receive any type of allogeneic blood transfusions, while all of control groups received allogeneic blood transfusions [48, 50, 54, 58, 70]. Finally, 13 studies with 14 comparisons including 544 participants were included in pooled analysis [40–42, 47, 49, 60, 61, 66–69]. The blood transfusion volume was reduced by 242.5 ml in the intervention group compared with control group (95% CI − 302.5 to − 182.6; *P* < 0.01; *I*^2^ = 95%; low quality of evidence, Fig. 4). Subgroup analyses according to age groups, controlled MAP levels, types of surgeries, or different combinations of other blood conservative methods or hypotensive methods did not explain heterogeneity, except for “nitrates” group (*I*^2^ = 0); and significant differences were identified in all subgroups (*P* < 0.01) (Additional file 2: Figure S6-S10).

##### Serious adverse postoperative events

Six studies including 286 participants reported this outcome, and all results were zero [40, 46, 47, 50, 51, 60]. The durations of follow-up ranged from 24 h to 7 days after surgery or during hospital stay. Thirteen studies including 634 participants although did not explicitly observe adverse postoperative events, there was probably the occurrence of no serious adverse postoperative events according to pre- and postoperative indicators (e.g., coagulation index, liver or kidney function, cognitive function) or no drop-outs [41, 43, 44, 53–56, 58, 65, 67–70].

## Discussion

Our meta-analysis shows the occurrence of no death or serious adverse postoperative events associated with deliberate hypotension in the included studies reporting or implying these outcomes. Furthermore, the use of deliberate hypotension may reduce intraoperative blood loss and blood transfusion volume during orthopedic surgery, irrespective of age groups, controlled MAP levels, types of surgeries, different combinations of other blood conservative methods, or hypotensive methods used.

### The safety consideration of using deliberate hypotension in orthopedic surgeries

A systematic review published in 2007 which included patients with orthognathic and orthopedic surgeries and described adverse events associated with deliberate hypotension reported the occurrence of no serious complications or death [8]. Orthognathic surgery is excluded in our analysis, as it is quite different from orthopedic surgery in terms of risk of death, intraoperative bleeding, and adverse postoperative events. Pooling the patient with orthognathic and orthopedic surgeries together may introduce significant heterogeneity. A recent observational study including 174 patients with the resection of pelvic and sacral tumors and a potential risk of intraoperative major blood loss assessed the safety of deliberate hypotension, and showed no apparent increase in serious adverse postoperative events and 90-day mortality [71]. A retrospective study examined the use of hypotensive epidural anesthesia in high-risk patients with preoperative renal dysfunction undergoing total hip arthroplasty, and found that hypotension per se, when carefully managed, did not predispose patients with chronic renal dysfunction to acute renal failure after surgery [72].

However, it is still unclear whether or not the use of deliberate hypotension is really safe, as there have been only very few studies reporting mortality and the occurrence of serious adverse postoperative events associated with deliberate hypotension. It has been shown that during the 1950s to 1960s, mortality associated with deliberate hypotension is 0.10% to 0.34% [10, 11]. With the introduction of new hypotensive agents or methods, generalization of advanced monitoring techniques, and combined use of other blood conservative measures in modern clinical practice, safety of deliberate hypotension would have been further improved. This means that assessment on the adverse outcomes associated with deliberate hypotension may require well designed RCT with an extremely large sample size. Another reason for the occurrence of no serious adverse postoperative events in our included studies may be the enrolment of relatively healthy patients (ASA I and II). In fact, different ages, controlled MAP levels, types of orthopedic surgeries, various combinations of other blood conservative measures, or hypotensive methods used may put patients using deliberate hypotension at different risks of mortality and morbidity. Due to limited number of studies and the occurrence of zero events in available literatures, however, subgroup analyses according to these heterogenetic factors seem impossible.

Although the mortality and serious adverse postoperative events are rare, clinical use of deliberate hypotension still needs caution. In clinical practice, it seems more reasonable to control MAP within a certain percentage of the baseline as to individual conditions of patients, rather than a specific value, especially for patients with hypertension and cardiovascular diseases. Most important, moreover, tissue hypoxia caused by hypotension should not be only limited to the vital organs such as heart, brain, and kidney. A logistic study has shown that hypotension can increase the incidence of postoperative nausea and vomiting [73], which may be related to the imbalance of oxygen supply and demand in the gastrointestinal tissues. Undoubtedly, nausea and vomiting will prolong hospital stay and reduce patients’ satisfaction. Another problem is that most of patients undergoing orthopedic surgery are elderly patients with a high risk of postoperative cognitive dysfunction (POCD) [74]. Some studies have observed the effect of deliberate hypotension on POCD [43, 44, 47, 55, 56, 65], but most of them did not use an internationally acknowledged measurement methods, which should include a validated battery of neuropsychological tests to assess global cognitive status, memory, attention, concentration, psychomotor skills, and others [75]. Thus, further studies are needed to observe the effect of deliberate hypotension on the occurrence of POCD in elderly patients undergoing orthopedic surgery.

It is generally believed that combining deliberate hypotension with other blood conservative methods can further reduce allogeneic blood transfusion. In fact, modern blood-sparing strategies also emphasize a combination of various methods. Hypovolemic hemodilution can increase cardiac output [76] and compensate for potential insufficient perfusion caused by decreased blood pressure; cell salvage can reduce allogeneic blood transfusion [50, 54]; tranexamic acid can reduce the bleeding [77]. However, whether combination of these methods with deliberate hypotension is safer than alone use of deliberate hypotension still need further investigation.

### Blood-sparing effect of deliberate hypotension

In previous study [8], our meta-analysis proves that the use of deliberate hypotension may reduce the blood loss and blood transfusion volume during orthopedic surgery. However, there is a high heterogeneity among studies reporting these outcomes. Furthermore, prespecified subgroup analyses mostly do not explain heterogeneity among studies. The experience of surgeons, different transfusion trigger points, various methods of measuring intraoperative blood loss, and within-subgroup heterogeneity (e.g., different types of surgeries) may explain this. Anyway, direction of the effect for almost all subgroups was consistent. From the perspective of sparing blood, deliberate hypotension is still a desirable technique for orthopedic surgery.

### Quality of evidence

The studies included in our analysis were at varying risks of bias and the evidence for outcomes was drawn from RCTs mostly at unclear and high risk of bias. Other than one [49], all of the included studies were justified as “unclear” or “high” risk study, mainly due to no sufficient information on randomization, allocation concealment, and blinding. Although all studies were reported to be randomized, methods of randomization were only described in few studies. Therefore, whether these studies have used “real” randomization is still doubtful. Fourteen studies [41, 43, 44, 46, 50, 52, 55–57, 60, 63, 65, 68, 69] did not describe the transfusion trigger points and were not blind to the personnels; this may allow the anesthesiologists to make a decision on blood transfusion only based on personal preference, subjective judgment, or surgeon’s demand, resulting in performance bias. Similarly, only 13 studies [41, 42, 46, 48, 49, 51, 53, 57, 58, 60, 63, 64, 67] mentioned the details of measuring intraoperative blood loss. Different measurement methods per se can result in a significant heterogeneity among studies; if without any specified measurement method, a detection bias cannot be avoided. In two studies, all of prespecified outcomes were not reported, though these outcomes were not primary or secondary outcomes in our analysis [42, 45]. In three studies, blood transfusion data were reported incompletely, which could not be included in our analysis [53, 63, 64]. Although a reporting bias may exist in these studies, the final results may probably not be influenced by lacking of these data. Due to limited number of studies included in our analysis, the funnel plot cannot be obtained and a possible publication bias cannot be excluded.

## Limitations

There are some limitations in our analysis. First, subgroup analyses were performed based on possible heterogeneity, but grouping a subgroup was relatively arbitrary, which might have resulted in the heterogeneity within subgroups. Furthermore, experience of surgeons, different transfusion trigger points, various methods of measuring intraoperative blood loss, and within-subgroup heterogeneity (e.g., different types of surgeries) may also introduce heterogeneity. Second, for multiple-comparison studies, the “shared” group was split with similar sample size to create two comparisons. A unit-of-analysis error may occur accordingly, even though this can facilitate investigation of heterogeneity and subgroup analyses. Third, our analysis only included the orthopedic surgeries in supine, lateral, and prone positions. For orthopedic surgeries requiring deliberate hypotension under other special position, such as shoulder arthroscopic surgery with a beach chair position, whether deliberate hypotension will bring additional risk and to what extent MAP level should be controlled are need further studies.

## Conclusions

Based on available evidence, it is still unclear whether or not deliberate hypotension is really safe for orthopedic surgery due to limited studies with very small sample size. However, deliberate hypotension may decrease intraoperative blood loss and blood transfusion volume irrespective of ages, controlled MAP levels, types of surgeries, hypotensive methods, or different combinations of other blood conservation strategies. From the perspective of sparing blood, deliberate hypotension is still a desirable technique for orthopedic surgery. The high-quality evidence from large well-designed RCTs is still needed to clarify the safety of this blood conservation technique for orthopedic surgery.

## Supplementary information

**Additional file 1:** The search strategy of four electronic databases.

**Additional file 2: Table S1.** Definition of outcomes. **Table S2.** Data collection form. **Table S3.** Risk of bias assessment of 31 included RCTs. **Table S4.** The GRADE for all outcomes.

**Additional file 3: Figure S1.** Forest plot for comparison of intraoperative blood loss based on different age groups between deliberate hypotension and no deliberate hypotension. IV, Inverse Variance. **Figure S2.** Forest plot for comparison of intraoperative blood loss based on different controlled MAP levels between deliberate hypotension and no deliberate hypotension. IV, Inverse Variance. **Figure S3.** Forest plot for comparison of intraoperative blood loss based on types of orthopedic surgeries between deliberate hypotension and no deliberate hypotension. IV, Inverse Variance. **Figure S4.** Forest plot for comparison of intraoperative blood loss based on different combinations of other blood conservative method between deliberate hypotension and no deliberate hypotension. IV, Inverse Variance; DH: deliberate hypotension; C: control; ANH: acute normovolemic hemodilution; AHH: acute hypervolemic hemodilution; A: autologous blood transfusion with cell salvage. **Figure S5.** Forest plot for comparison of intraoperative blood loss based on different hypotensive methods between deliberate hypotension and no deliberate hypotension. IV, Inverse Variance. **Figure S6.** Forest plot for comparison of blood transfusion volume based on different age groups between deliberate hypotension and no deliberate hypotension. IV, Inverse Variance. **Figure S7.** Forest plot for comparison of blood transfusion volume based on different controlled MAP levels between deliberate hypotension and no deliberate hypotension. IV, Inverse Variance. **Figure S8.** Forest plot for comparison of blood transfusion volume based on different types of orthopedic surgeries between deliberate hypotension and no deliberate hypotension. IV, Inverse Variance. **Figure S9.** Forest plot for comparison of blood transfusion volume based on different combinations of other blood conservative method between deliberate hypotension and no deliberate hypotension. IV, Inverse Variance; DH: deliberate hypotension; C: control; ANH: acute normovolemic hemodilution; AHH: acute hypervolemic hemodilution; A: autologous blood transfusion with cell salvage. **Figure S10.** Forest plot for comparison of blood transfusion volume based on different hypotensive methods between deliberate hypotension and no deliberate hypotension. IV, Inverse Variance.

## Data Availability

All data generated or analyzed during this study are included in this published article.

## Abbreviations

CI: Confidence interval
IQR: Interquartile range
MAP: Mean artery pressure
RCTs: Randomized controlled trials
SD: Standard deviation
