# Supplementary material for: Is deliberate hypotension a safe technique for orthopedic surgery?: a systematic review and meta-analysis of parallel randomized controlled trials
Source: J Orthop Surg Res. 2019 Dec 2;14:409. doi: 10.1186/s13018-019-1473-6 (PMC6889611; doi:10.1186/s13018-019-1473-6)
Supplement: Supplementary file 1 — Additional file 1: The search strategy of four electronic databases. [file 13018_2019_1473_MOESM1_ESM.docx]

**The search strategy of four electronic databases**

***A. Cochrane Library***

#1MeSH descriptor: [Hypotension, Controlled] explode all trees (195)

#2((deliberate or induced or controlled or an?esthesia) near/2 hypotens*):ti,ab,kw (Word variations have been searched) (821)

#3#1 or #2 (821)

#4MeSH descriptor: [Orthopedic Procedures] explode all trees (11214)

#5MeSH descriptor: [Orthopedics] explode all trees (345)

#6(ort?op?ed* near/5 (surg* or operat* or patient*)):ti,ab,kw (Word variations have been searched) (1310)

#7#4 or #5 or #6 (12413)

#8#3 and #7 in Trials (61)

***B. MEDLINE***

1 exp Hypotension, Controlled/ or ((deliberate or induced or controlled or an?esthesia) adj2 hypotens$).mp. (6487)

2 exp Orthopedic Procedures/ or exp Orthopedics/ or (ort?op?ed$ adj5 (surg$ or operat$ or patient$)).mp. (260062)

3 ((randomized controlled trial or controlled clinical trial).pt. or randomized.ab. or placebo.ab. or drug therapy.fs. or randomly.ab. or trial.ab. or groups.ab.) not (exp animals/ not humans.sh.) (3164676)

4 1 and 2 and 3 (96)

***C. EMBASE***

1 ((deliberate or induced or controlled or an?esthesia) adj2 hypotens$).mp. (7525)

2 exp orthopedic surgery/ or (ort?op?ed$ adj5 (surg$ or operat$ or patient$)).mp. (388871)

3 exp randomized-controlled-trial/ or exp double-blind-procedure/ or exp crossover-procedure/ or exp single-blind-procedure/ or exp randomization/ or ((clin$ adj2 trial) or ((singl$ or doubl$ or trebl$ or tripl$) adj (blind$ or mask$)) or (random$ adj5 (assign$ or allocat$)) or (randomi$ or crossover)).mp. (1620802)

4 1 and 2 and 3 (107)

***D. CINAHL***

S1 TX ((deliberate or induced or controlled or an?esthesia) n2 hypotens*) (1,547)

S2 TX ( (ort?op?ed* n5 (surg* or operat* or patient*)) ) OR MH "Orthopedic Surgery+" (95,584)

S3 MM "Clinical Trials+" OR MH triple-blind studies OR MH double-blind studies OR MH single-blind studies OR MH "Random Assignment" OR TX (clin* N2 trial*) or TX (randomi* control* trial*) or TX (random* N1 (allocat* OR assign*)) or TX (randomi* or crossover) or TX ((singl* n1 blind*) or (singl* n1 mask*)) or TX ((doubl* n1 blind*) or (doubl* n1 mask*)) or TX ((tripl* n1 blind*) or (tripl* n1 mask*)) or TX ((trebl* n1 blind*) or (trebl* n1 mask*)) (1,025,446)

S4 S1 AND S2 AND S3 (42)

***E. ISI WEB OF SCIENCE INCLUDING BIOSIS PREVIEWS AND BIOSIS CITATION INDEX***

# 1 TOPIC**:** ((clin* Near/2 trial*) or (randomi* control* trial*) or (random* Near/1 (allocat* OR assign*)) or (randomi* or crossover) or ((singl* Near/1 blind*) or (singl* Near/1 mask*)) or ((doubl* Near/1 blind*) or (doubl* Near/1 mask*)) or ((tripl* Near/1 blind*) or (tripl* Near/1 mask*)) or ((trebl* Near/1 blind*) or (trebl* Near/1 mask*))) *Timespan=All years Search language=Auto* (Approximately 3,859,383)

# 2 TOPIC: (((deliberate or induced or controlled or anaesthesia or anesthesia) near/2 hypotens*)) Timespan=All years Search language=Auto **(**Approximately 28,643)

# 3 TOPIC: (((orthoped* or Orthiped*) near/5 (surg* or operat* or patient*))) Timespan=All years Search language=Auto (Approximately 101,347)

# 4 #3 AND #2 AND #1 Timespan=All years Search language=Auto (32**)**

***F. SCIENCEDIRECT***

(TITLE-ABSTR-KEY((orthoped* W/5 surg*) or (orthoped* W/5 operat*) or (orthoped* W/5 patient*) or (Orthiped* W/5 surg*) or (Orthiped* W/5 operat*) or (Orthiped* W/5 patient*))) AND (TITLE-ABSTR-KEY((deliberate W/2 hypotens*) or (induced W/2 hypotens*) or (controlled W/2 hypotens*) or (anaesthesia W/2 hypotens*) or (anesthesia W/2 hypotens*))) AND (TITLE-ABSTR-KEY((clin* W/2 trial*) or (random* W/1 allocat*) OR (random* W/1 assign*) or randomi* or crossover ) or TITLE-ABSTR-KEY((singl* W/1 blind*) or (singl* W/1 mask*) or (doubl* W/1 blind*) or (doubl* W/1 mask*) or (tripl* W/1 blind*) or (tripl* W/1 mask*) or (trebl* W/1 blind*) or (trebl* W/1 mask*)))
*[All Sources(- All Sciences -)]* (8)

***G. HMIC***

1 ((deliberate or induced or controlled or an?esthesia) adj2 hypotens$).mp. (1)

2 (ort?op?ed$ adj5 (surg$ or operat$ or patient$)).mp. (568)

3 1 and 2 (0)

***H. OPENGREY***

deliberate NEAR/2 hypotens* (0)

induced NEAR/2 hypotens* (5)

controlled NEAR/2 hypotens* (1)

anaesthesia NEAR/2 hypotens* (1)

anesthesia NEAR/2 hypotens* (3)

***I***. *www.clinicaltrials.com*

Hypotension and orthopedic = 13

***J. www.controlled-trials.com/***

orthopedic hypotension random = 0

orthopedic hypotension randomised = 2

orthopedic hypotension randomized = 2

orthipedic hypotension random = 0

orthipedic hypotension randomised = 0

orthipedic hypotension randomized = 0

***K. CNKI***

#1 主题=血液稀释

#2 主题=血液回收

#3 主题=血液黏度

#4 主题=血量

#5 主题=体外循环

#6 主题=血浆代用品

#7 主题=控制性降压

#8 主题=降压控制

#9 主题=控制血压

#10 主题=低血压

#11 主题=自体血

#12 OR/1-11

#13 主题=脊柱矫正

#14 主题=骨科手术

#15 主题=矫形外科手术

#16 主题=截肢术

#17 主题=关节离断

#18 主题=骨盆切除

#19 主题=关节融合

#20 主题=椎间盘化学松解

#21 主题=椎间盘松解

#22 主题=椎体成形

#23 主题=脊柱融合

#24 主题=关节成形

#25 主题=骨延长术

#26 主题=伊利扎罗夫

#27 主题=骨生成

#28 主题=骨折固定

#29 主题=截骨术

#30 主题=牵引术

#31 主题=骨移植

#32 主题=骨组织移植

#33 主题=椎间盘切除

#34 主题=四肢救助

#35 主题=腱固定术

#36 主题=椎体成形术

#37 主题=骨科矫形

#38 主题=骨科矫正

#39 主题=脊柱侧弯矫正

#40 OR/14-39

#41 全文=随机

#42 AND/13,40,41

***L. CBM***

#1 "矫形外科手术"[不加权:扩展]

#2 "截肢术"[不加权:扩展]

#3 "关节离断术"[不加权:扩展]

#4 "偏侧骨盆切除术"[不加权:扩展]

#5 "关节融合术"[不加权:扩展]

#6 "椎间盘化学松解术"[不加权:扩展]

#7 "椎体成形术"[不加权:扩展]

#8 "脊柱融合术"[不加权:扩展]

#9 "关节成形术"[不加权:扩展]

#10 "关节成形术, 置换"[不加权:扩展]

#11 "关节成形术, 置换, 髋"[不加权:扩展]

#12 "关节成形术, 置换, 膝"[不加权:扩展]

#13 "关节成形术, 置换, 指"[不加权:扩展]

#14 "关节成形术, 软骨下"[不加权:扩展]

#15 "关节镜检查"[不加权:扩展]

#16 "骨延长术"[不加权:扩展]

#17 "伊利扎罗夫技术"[不加权:扩展]

#18 "骨生成, 牵张"[不加权:扩展]

#19 "骨折固定术"[不加权:扩展]

#20 "骨折固定术, 内"[不加权:扩展]

#21 "骨折固定术, 髓内"[不加权:扩展]

#22 "截骨术"[不加权:扩展]

#23 "截骨术, 勒福"[不加权:扩展]

#24 "牵引术"[不加权:扩展]

#25 "骨移植"[不加权:扩展]

#26 "骨-髌韧带-骨组织移植重建术"[不加权:扩展]

#27 "椎间盘切除术"[不加权:扩展]

#28 "椎间盘切除术, 经皮"[不加权:扩展]

#29 "四肢救助"[不加权:扩展]

#30 "腱固定术"[不加权:扩展]

#31 "椎体成形术"[不加权:扩展]

#32 "骨科手术"[常用字段:智能]

#33 "矫形外科手术"[常用字段:智能]

#34 "截肢术"[常用字段:智能]

#35 "关节离断"[常用字段:智能]

#36 "骨盆切除"[常用字段:智能]

#37 "关节融合"[常用字段:智能]

#38 "椎间盘化学松解"[常用字段:智能]

#39 "椎间盘松解"[常用字段:智能]

#40 "椎体成形"[常用字段:智能]

#41 "脊柱融合"[常用字段:智能]

#42 "关节成形"[常用字段:智能]

#43 "骨延长术"[常用字段:智能]

#44 "伊利扎罗夫"[常用字段:智能]

#45 "骨生成"[常用字段:智能]

#46 "骨折固定"[常用字段:智能]

#47 "截骨术"[常用字段:智能]

#48 "牵引术"[常用字段:智能]

#49 "骨移植"[常用字段:智能]

#50 "骨组织移植"[常用字段:智能]

#51 "椎间盘切除"[常用字段:智能]

#52 "四肢救助"[常用字段:智能]

#53 "腱固定术"[常用字段:智能]

#54 "椎体成形术"[常用字段:智能]

#55 "骨科矫形"[常用字段:智能]

#56 "骨科矫正"[常用字段:智能]

#57 "脊柱侧弯矫正"[常用字段:智能]

#58 "脊柱矫正"[常用字段:智能]

#59 OR/1-58

#60 "血液稀释"[不加权:扩展]

#61"血液稀释"[常用字段:智能]

#62"血液回收"[常用字段:智能]

#63"血液黏度"[常用字段:智能]

#64"血量"[常用字段:智能]

#65"体外循环"[常用字段:智能]

#66"血浆代用品"[常用字段:智能]

#67"控制性降压"[常用字段:智能]

#68"降压控制"[常用字段:智能]

#69"控制血压"[常用字段:智能]

#70"低血压"[常用字段:智能]

#71"自体血"[常用字段:智能]

#72 OR/60-71

#73 "随机"[全字段:智能]

#74 "随机对照试验(主题)"[不加权:扩展]

#75 "随机对照试验"[不加权:扩展]

#76 OR/73-75

#78 AND/59,72,76

***M. Wanfang***

#1 主题:(血液稀释)

#2 主题:(血液回收)

#3 主题:(血液黏度)

#4 主题:(血量)

#5 主题:(体外循环)

#6 主题:(血浆代用品)

#7 主题:(控制性降压)

#8 主题:(降压控制)

#9 主题:(控制血压)

#10 主题:(低血压)

#11 主题:(自体血)

#12 OR/1-11

#13 主题:(脊柱矫正)

#14 主题:(骨科手术)

#15 主题:(矫形外科手术)

#16 主题:(截肢术)

#17 主题:(关节离断)

#18 主题:(骨盆切除)

#19 主题:(关节融合)

#20 主题:(椎间盘化学松解)

#21 主题:(椎间盘松解)

#22 主题:(椎体成形)

#23 主题:(脊柱融合)

#24 主题:(关节成形)

#25 主题:(骨延长术)

#26 主题:(伊利扎罗夫)

#27 主题:(骨生成)

#28 主题:(骨折固定)

#29 主题:(截骨术)

#30 主题:(牵引术)

#31 主题:(骨移植)

#32 主题:(骨组织移植

#33 主题:(椎间盘切除

#34 主题:(四肢救助)

#35 主题:(腱固定术)

#36 主题:(椎体成形术)

#37 主题:(骨科矫形)

#38 主题:(骨科矫正)

#39 主题:(脊柱侧弯矫正)

#40 OR/14-39

#41 全部:(随机)

#42 AND/13,40,41

***N. VIP***

#1 M=血液稀释

#2 M=血液回收

#3 M=血液黏度

#4 M=血量

#5 M=体外循环

#6 M=血浆代用品

#7 M=控制性降压

#8 M=降压控制

#9 M=控制血压

#10 M=低血压

#11 M=自体血

#12 OR/1-11

#13 M=脊柱矫正

#14 M=骨科手术

#15 M=矫形外科手术

#16 M=截肢术

#17 M=关节离断

#18 M=骨盆切除

#19 M=关节融合

#20 M=椎间盘化学松解

#21 M=椎间盘松解

#22 M=椎体成形

#23 M=脊柱融合

#24 M=关节成形

#25 M=骨延长术

#26 M=伊利扎罗夫

#27 M=骨生成

#28 M=骨折固定

#29 M=截骨术

#30 M=牵引术

#31 M=骨移植

#32 M=骨组织移植

#33 M=椎间盘切除

#34 M=四肢救助

#35 M=腱固定术

#36 M=椎体成形术

#37 M=骨科矫形

#38 M=骨科矫正

#39 M=脊柱侧弯矫正

#40 OR/14-39

#41 U=随机

#42 AND/13,40,41
