# Supplementary material for: Is deliberate hypotension a safe technique for orthopedic surgery?: a systematic review and meta-analysis of parallel randomized controlled trials
Source: J Orthop Surg Res. 2019 Dec 2;14:409. doi: 10.1186/s13018-019-1473-6 (PMC6889611; doi:10.1186/s13018-019-1473-6)
Supplement: Supplementary file 2 — Additional file 2: Table S1. Definition of outcomes. Table S2. Data collection form. Table S3. Risk of bias assessment of 31 included RCTs. Table S4. The GRADE for all outcomes. [file 13018_2019_1473_MOESM2_ESM.docx]

**Table S1 Definition of outcomes**

| **Outcomes** | **Definition** |
| --- | --- |
| Overall mortality | All causes: the data available in each RCT within 28 days after surgery was used. |
| Volume of blood transfused | The total volume of intraoperative blood transfusion. The blood transfusion referred to the transfusion of any type of allogeneic blood product. |
| Intraoperative blood loss | Measured by recording the volume of blood collected in the suction bottles, and the increased weight of gauzes. When the cell salvage was used during operation, the intraoperative blood loss was measured by recording the volume of blood collected in the suction bottles and the cell salvage, and the increased weight of gauze |
| Occurrence of serious adverse events | Serious adverse events included severe cerebral, cardiac, renal, hepatic, and hematologic complications. Study authors' own definitions of these serious adverse events should be eligible clinically and the data before hospital discharge in each RCT was used. If this was not available, the data within 28 days after surgery was used.  Serious cerebral complications were defined as cerebral ischemia or cerebral stroke diagnosed or suspected by using study authors' own definition (disturbance of consciousness with Glasgow score lower than eight, computed tomography (CT), magnetic resonance imaging (MRI), etc).  Serious cardiac complications were defined as myocardial ischemia, myocardial infarction, pulmonary edema, or heart failure diagnosed or suspected by using study authors' own definition (creatine kinase isoenzyme (CK-MB) level, electrocardiographic changes, chest radiograph, etc).  Serious renal complications were defined as renal dysfunction or renal failure diagnosed or suspected by using study authors' own definition (serum creatinine level, urine output, etc.).  Serious hepatic complications were defined as liver dysfunction or liver failure diagnosed or suspected by using study authors' own definition (serum transaminase level, etc.).  Severe hematologic complications were defined as severe coagulation dysfunction and thromboembolism diagnosed or suspected by using study authors' own definition (coagulation tests, ultrasound, etc.). |

| **Table S2 Data extraction form** | | | | | | | | | |
| --- | --- | --- | --- | --- | --- | --- | --- | --- | --- |
| **Study ID** (family name of 1^st^ author and year of publication and comparison number) | | | | | | | | | |
| **Date of data extraction** | | | | | | | | | |
| **Site of data extraction** | | | | | | | | | |
| **Name of person extracting data** | | | | | | | | | |
| **Date of completing data extraction** | | | | | | | | | |
| **Title** | | | | | | | | | |
| **Contact details (Email address)** | | | | | | | | | |
| **Source: electronic database/ unpublished information/ personal communications/ other** | | | | | | | | | |
| **Publication type** | | | | | | | | | |
| **Informed consent obtained:** **Yes** **No** **Unclear** | | | | | | | | | |
| **Ethical approval obtained :** **Yes No** **Unclear** | | | | | | | | | |
| **Study funding sources** | | | | | | | | | |
| **Possible conflicts of interest** | | | | | | | | | |
| **Notes** | | | | | | | | | |
| **Eligibility** | | | | | | | | | |
| **Characteristics** | | **Eligibility criteria** | | | | | | **Location in text** | |
|  | |  | | | Yes | No | Unclear |  | |
| **Type of study** | | The study was a randomized controlled trial without any significant ethical issues | | |  |  |  |  | |
| **Participants** | | The participants received an orthopedic surgery? | | |  |  |  |  | |
|  |  | The participants were free of any history of neurologic or psychiatric dysfunction, uncontrolled hypertension, ischemic heart disease, stroke, renal or hepatic dysfunction, severe peripheral vascular disease, uncorrected hypovolemia, or anemia (hemoglobin level≤110g/dl) | | |  |  |  |  | |
| **Intervention** | | The experimental group use deliberate hypotension within an acceptable limit | | |  |  |  |  | |
|  |  | The control group did not use deliberate hypotension | | |  |  |  |  | |
| **Types of outcome (Did the study report any one of)** | | Overall mortality | | |  |  |  |  | |
|  |  | Volume of blood transfused | | |  |  |  |  | |
|  |  | Intraoperative blood loss | | |  |  |  |  | |
|  |  | Occurrence of serious adverse events after surgery | | |  |  |  |  | |
| **If any of the above answers are ‘No’, do not proceed and make a justification as:**  Included  Excluded and should be listed in the excluded table  Excluded and should NOT be listed in the excluded table  More information needed before inclusion decision (contact the original author)  Reason for exclusion:  **If included, continue.** | | | | | | | | | |
| **Population and setting** | | | | | | | | | |
|  | **Intervention** | | | **Control** | | | **P value**  **(if any)** | | **Location in text** |
| **Number of participants (n)** |  | | |  | | |  | |  |
| **Number of drop-outs and the reason (n)** |  | | |  | | |  | |  |
| **Age (mean/SD)** |  | | |  | | |  | |  |
| **Sex (M/F, n)** |  | | |  | | |  | |  |
| **ASA grade (Ⅰ/Ⅱ/Ⅲ/Ⅳ, n)** |  | | |  | | |  | |  |
| **Co-morbidity** |  | | |  | | |  | |  |
| **Inclusion criteria** |  | | | | | | | |  |
| **Excluded criteria** |  | | | | | | | |  |
| **Type of surgery** |  | | | | | | | |  |
| **Type of anaesthesia** |  | | | | | | | |  |
| **Transfusion trigger method** |  | | | | | | | |  |
| **Notes** | | | | | | | | | |
| **Risk of Bias assessment** | | | | | | | | | |
| **Domain** | | **Risk of bias** | | | **Support for judgement** | | | | **Location in text** |
|  |  | Low | High | Unclear |  |  |  |  |  |
| **Random sequence generation** **(selection bias)** | |  |  |  |  | | | |  |
| **Allocation concealment** **(selection bias)** | |  |  |  |  | | | |  |
| **Blinding of participants and personnel** **(performance bias)** | |  |  |  |  | | | |  |
| **Blinding of outcome assessment** **(detection bias)** | |  |  |  |  | | | |  |
| **Incomplete outcome data** **(attrition bias)** | |  |  |  |  | | | |  |
| **Selective outcome reporting?** **(reporting bias)** | |  |  |  |  | | | |  |
| **Other bias** | |  |  |  |  | | | |  |
| **Notes:** | | | | | | | | | |
| **Intervention groups** | | | | | | | | | |
| **Comparison** | | | | | | | | | |
|  | | **Description** | | | | | | | **Location in text** |
|  |  | **Intervention** | | | **Control** | | | |  |
| **Total number** | |  | | |  | | | |  |
| **Method of deliberate hypotension** | |  | | |  | | | |  |
| **MAP level** | |  | | |  | | | |  |
| **Duration of deliberate hypotension** | |  | | |  | | | |  |
| **Whether combined with haemodilution or cell salvage and the detailed method** | |  | | |  | | | |  |
| **Notes:** | | | | | | | | | |
| **Outcomes** | | | | | | | | | |
| **Outcomes the study reported** | | | | | | | | | |
| **Outcome (number)**  (Repeat this section for each outcome) | | | | | | | | | |
|  | | **Description** | | | | | | | **Location in text** |
| **Outcome name** | |  | | | | | | |  |
| **Outcome definition** | |  | | | | | | |  |
| **Timing of measurement** | |  | | | | | | |  |
| **Statistics for measurement** | |  | | | | | | |  |
| **Unit of measurement** | |  | | | | | | |  |
| **Is outcome selectively reported?** | |  | | | | | | |  |
| **Notes:** | | | | | | | | | |
| **Results**  (Repeat the relative section for each outcome at each time point) | | | | | | | | | |
| **Dichotomous outcome or rare events** | | | | | | | | | |
|  | | **Description** | | | | | | | **Location in text** |
| **Outcome** | |  | | | | | | |  |
| **Time point** | |  | | | | | | |  |
| **Results** | | **Intervention** | | | **Control** | | | |  |
|  |  | Number of events | Number of participants or number of person-time at risk in each group | SE | Number of events | Number of participants or number of person-time at risk in each group | SE | |  |
|  |  |  |  |  |  |  |  | |  |
| **Number of drop-outs and the reason** | |  | | |  | | | |  |
| **Notes (**For the studies only report odds ratio or risk ratio that accompanied by 95% CI or an exact P value, the formulae used to transform them into SE): | | | | | | | | | |
| **Continuous outcome** | | | | | | | | | |
|  | | **Description** | | | | | | |  |
| **Outcome** | |  | | | | | | |  |
| **Time point** | |  | | | | | | |  |
| **Results** | | **Intervention** | | | **Control** | | | |  |
|  |  | Mean | SD (or other variance) | No. of participants | Mean | SD (or other variance) | No. of participants | |  |
|  |  |  |  |  |  |  |  | |  |
| **Number of drop-outs and the reason** | |  | | |  | | | |  |
| **Notes (**For the studies that report the average or variance instead of SD, the formulae use to transform them into SD): | | | | | | | | | |

**Table S3 Risk of bias assessment of 31 including RCTs**

| **Domains** | **Description** |
| --- | --- |
| Allocation (selection bias) | All included studies were reported to be randomized. Eight studies described the methods of sequence generation [40, 47, 49-51, 62, 67, 70]. Two studies used adequate allocation concealment of patient randomization [46, 49]. For other studies, authors did not describe the allocation concealment and there was insufficient information to judgement the risk of bias. |
| Blinding (performance bias and detection bias) | Although only one study blinded the participants and personnel [49], we judged that the outcomes would be not likely to be influenced by lack of blinding if a detailed transfusion trigger point was provided in method. Since the patients involved in our analysis were all surgical patients, who did not know their grouping; meanwhile, during anesthesia, it was impossible for their anesthesiologists not knowing the blood pressure of their patients. As long as there was a clear blood transfusion trigger point, anesthesiologist may not make a decision according to their own preference. Thus, 17 studies [40, 42, 45, 47-49, 51, 53, 54, 58, 59, 61, 62, 64, 66, 67, 70] were also judged as "low-risk” of bias for this domain; Only one study [49] blinded outcome assessment. For other included studies, authors did not describe the blinding and there was insufficient information to judge the risk of bias. |
| Incomplete outcome data (attrition bias) | There was no missing data in all included studies. |
| Selective reporting (reporting bias) | Five studies did not report all prespecified outcomes in results. One study [42] did not report the fluid infusion and urine output; one study [45] did not report the volume of fluid input; two studies [53, 64] were supposed to report the volume of blood transfused in results, however, only the ratio of allogeneic blood transfused or the number of participants with blood transfused were shown instead; Data on intraoperative blood loss in one study [63] reported no SD or P value, which could not be used in meta-analysis. |
| Other potential sources of bias | No other potential sources of bias. |

| **Outcomes** | **Illustrative comparative risks* (95% CI)** | | **Relative effect (95% CI)** | **No of Participants (studies)** | **Quality of the evidence (GRADE)** | **Comments** |
| --- | --- | --- | --- | --- | --- | --- |
|  | **Assumed risk** | **Corresponding risk** |  |  |  |  |
|  | **No deliberate hypotension** | **Deliberate hypotension** |  |  |  |  |
| **Overall mortality** Follow-up: more than 24 hours after surgery | See comment | See comment | Not estimable | 120 (2 studies) | ⊕⊕⊝⊝ **low**^1^ | 0 participants had event of death. |
| **Volume of blood transfused** | Not estimable | The mean volume of blood transfused in the intervention groups was **242.53 lower** (302.48 to 182.58 lower) | Not estimable | 544 (13 studies) | ⊕⊕⊝⊝ **low**^2^ |  |
| **Intraoperative blood loss** | Not estimable | The mean intraoperative blood loss in the intervention groups was **376.71 lower** (428.12 to 325.3 lower) | Not estimable | 1398 (29 studies) | ⊕⊕⊝⊝ **low**^2^ |  |
| **Occurrence of serious adverse events after surgery** Follow-up: more than 24 hours after surgery | See comment | See comment | Not estimable | 286 (6 studies) | ⊕⊕⊝⊝ **low**^1^ | 0 participants had serious adverse events after surgery. |
| **Length of hospital stay** | See comment | See comment | See comment | See comment | See comment | No studies reported this outcome. |
| *The basis for the **assumed risk** (e.g. the median control group risk across studies) is provided in footnotes. The **corresponding risk** (and its 95% confidence interval) is based on the assumed risk in the comparison group and the **relative effect** of the intervention (and its 95% CI). **CI:** Confidence interval; | | | | | | |
| GRADE Working Group grades of evidence **High quality:** we are very confident that the true effect lies close to that of the estimate of the effect; **Moderate quality:** we are moderately confident in the effect estimate; the true effect is likely to be close to the estimate of effect, but there is a possibility that it is substantially different; **Low quality:** our confidence in the effect estimate is limited; the true effect may be substantially different from the estimate of the effect; **Very low quality:** we have very little confidence in the effect estimate; the true effect is likely to be substantially different from the estimate of effect. | | | | | | |

**Table S4 The GRADE for all outcomes**

^1^ Downgraded twice for imprecision due to very small sample size.
^2^ Downgraded twice for inconsistency due to high level heterogeneity.窗体顶端

窗体底端
