# Supplementary material for: Is deliberate hypotension a safe technique for orthopedic surgery?: a systematic review and meta-analysis of parallel randomized controlled trials
Source: J Orthop Surg Res. 2019 Dec 2;14:409. doi: 10.1186/s13018-019-1473-6 (PMC6889611; doi:10.1186/s13018-019-1473-6)
Supplement: Supplementary file 3 — Additional file 3: Figure S1. Forest plot for comparison of intraoperative blood loss based on different age groups between deliberate hypotension and no deliberate hypotension. IV, Inverse Variance. Figure S2. Forest plot for comparison of intraoperative blood loss based on different controlled MAP levels between deliberate hypotension and no deliberate hypotension. IV, Inverse Variance. Figure S3. Forest plot for comparison of intraoperative blood loss based on types of orthopedic surgeries between deliberate hypotension and no deliberate hypotension. IV, Inverse Variance. Figure S4. Forest plot for comparison of intraoperative blood loss based on different combinations of other blood conservative method between deliberate hypotension and no deliberate hypotension. IV, Inverse Variance; DH: deliberate hypotension; C: control; ANH: acute normovolemic hemodilution; AHH: acute hypervolemic hemodilution; A: autologous blood transfusion with cell salvage. Figure S5. Forest plot for comparison of intraoperative blood loss based on different hypotensive methods between deliberate hypotension and no deliberate hypotension. IV, Inverse Variance. Figure S6. Forest plot for comparison of blood transfusion volume based on different age groups between deliberate hypotension and no deliberate hypotension. IV, Inverse Variance. Figure S7. Forest plot for comparison of blood transfusion volume based on different controlled MAP levels between deliberate hypotension and no deliberate hypotension. IV, Inverse Variance. Figure S8. Forest plot for comparison of blood transfusion volume based on different types of orthopedic surgeries between deliberate hypotension and no deliberate hypotension. IV, Inverse Variance. Figure S9. Forest plot for comparison of blood transfusion volume based on different combinations of other blood conservative method between deliberate hypotension and no deliberate hypotension. IV, Inverse Variance; DH: deliberate hypotension; C: control; ANH: acute normo [file 13018_2019_1473_MOESM3_ESM.docx]

**Additional file 3: Supplemental Figures 1-10.**


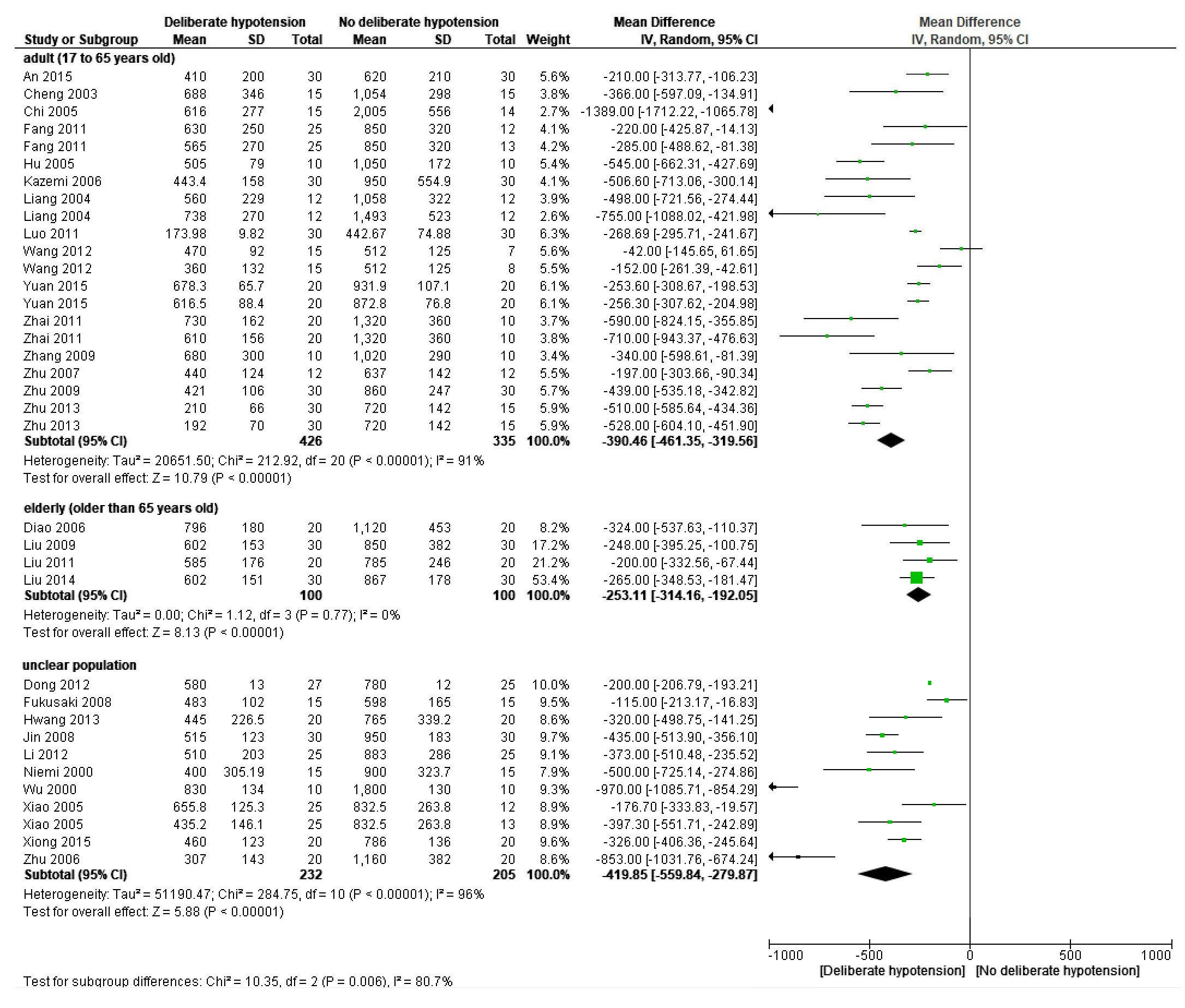


**Fig. S1** Forest plot for comparison of intraoperative blood loss based on different age groups between deliberate hypotension and no deliberate hypotension. IV, Inverse Variance.


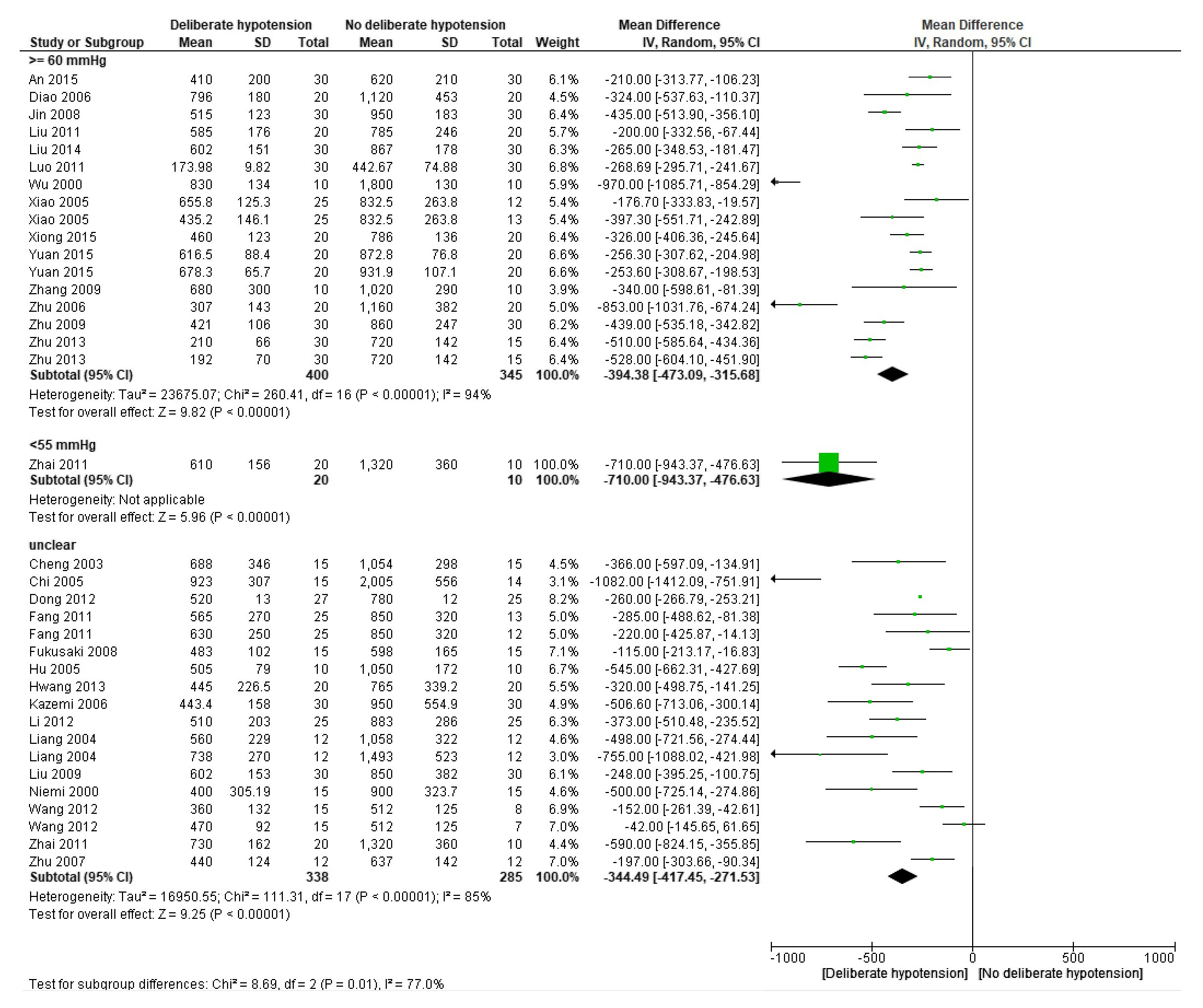


**Fig. S2** Forest plot for comparison of intraoperative blood loss based on different controlled MAP levels between deliberate hypotension and no deliberate hypotension. IV, Inverse Variance.


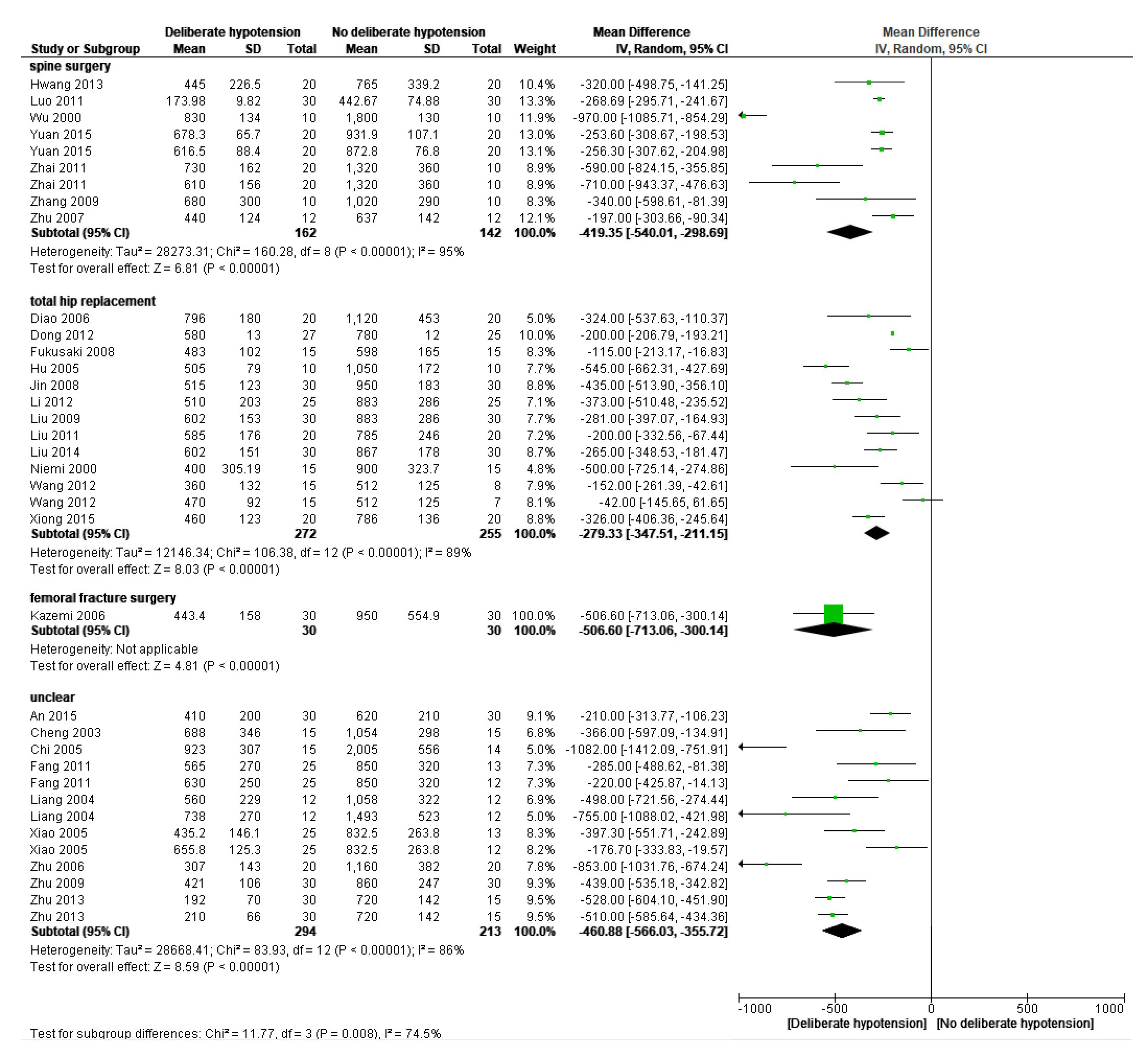


**Fig. S3** Forest plot for comparison of intraoperative blood loss based on types of orthopedic surgeries between deliberate hypotension and no deliberate hypotension. IV, Inverse Variance.


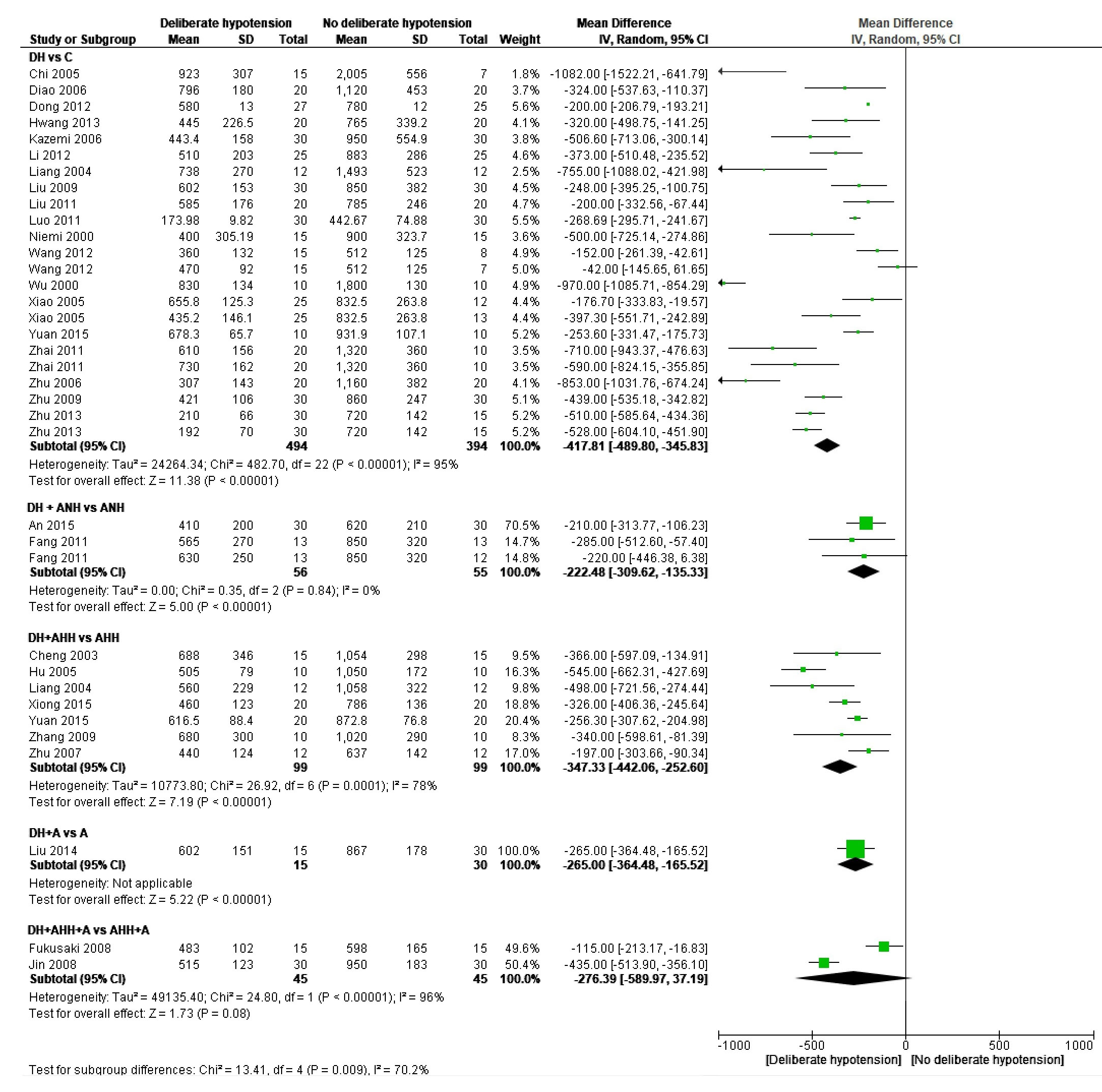


**Fig. S4** Forest plot for comparison of intraoperative blood loss based on different combinations of other blood conservative method between deliberate hypotension and no deliberate hypotension. IV, Inverse Variance; DH: deliberate hypotension; C: control; ANH: acute normovolemic hemodilution; AHH: acute hypervolemic hemodilution; A: autologous blood transfusion with cell salvage.


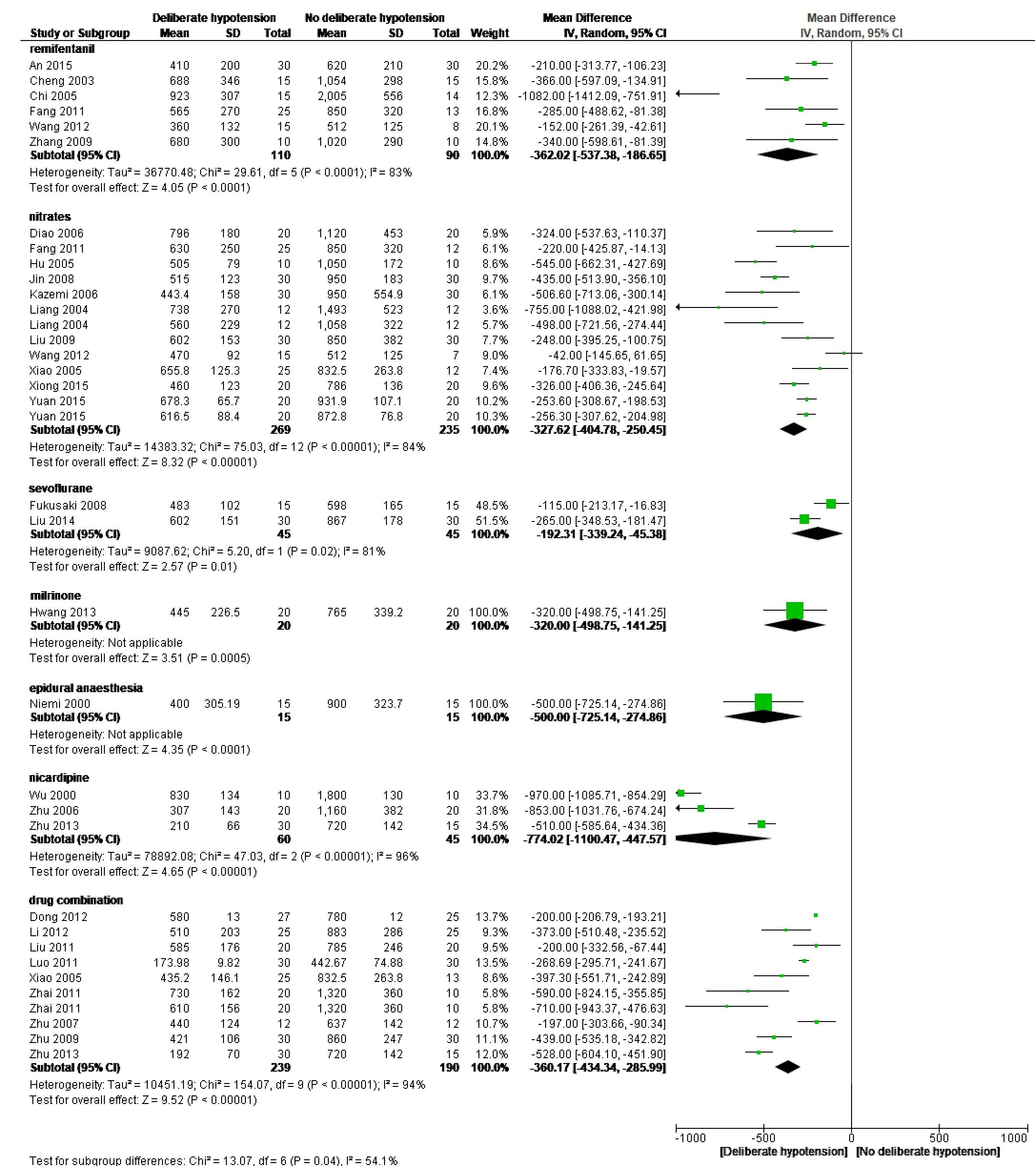


**Fig. S5** Forest plot for comparison of intraoperative blood loss based on different hypotensive methods between deliberate hypotension and no deliberate hypotension. IV, Inverse Variance.


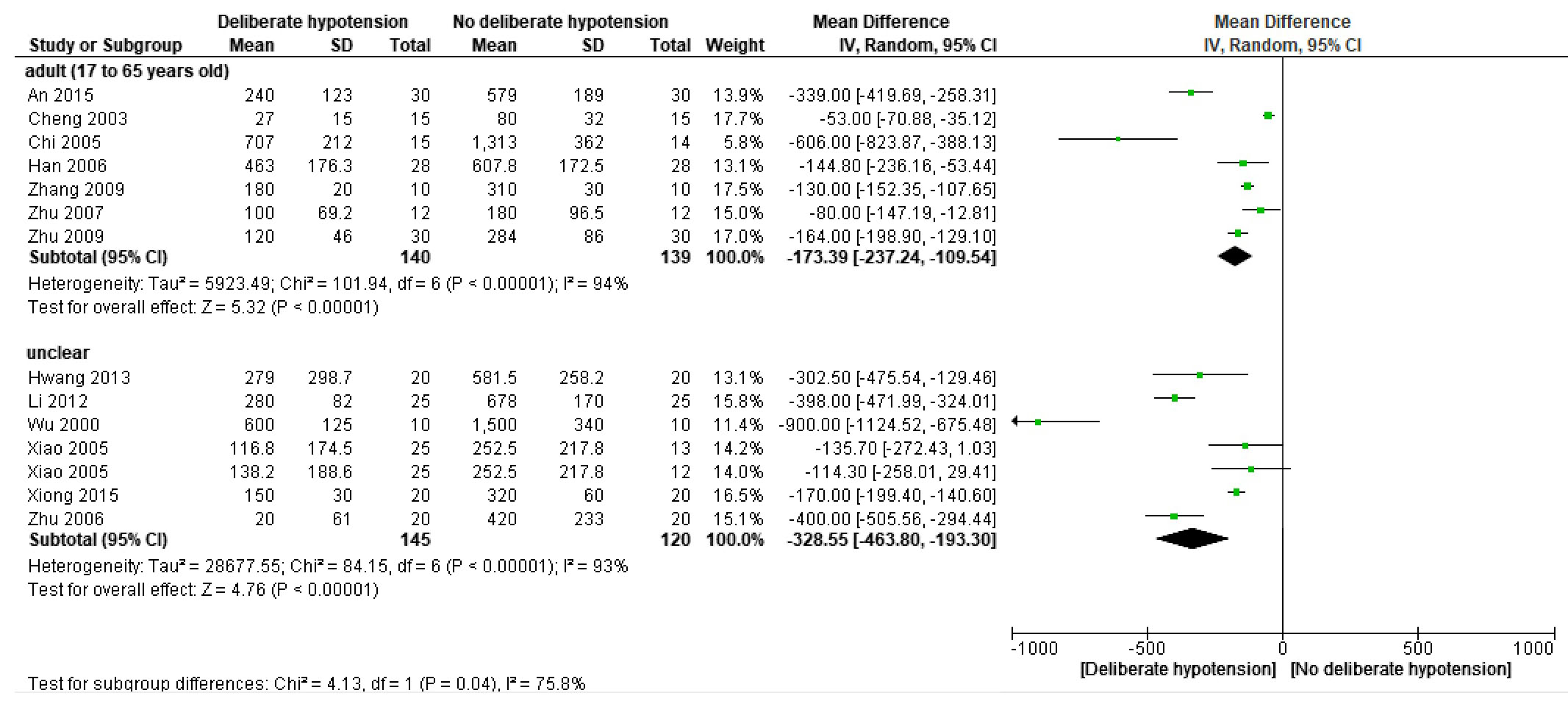


**Fig. S6** Forest plot for comparison of volume of blood transfused based on different age groups between deliberate hypotension and no deliberate hypotension. IV, Inverse Variance.


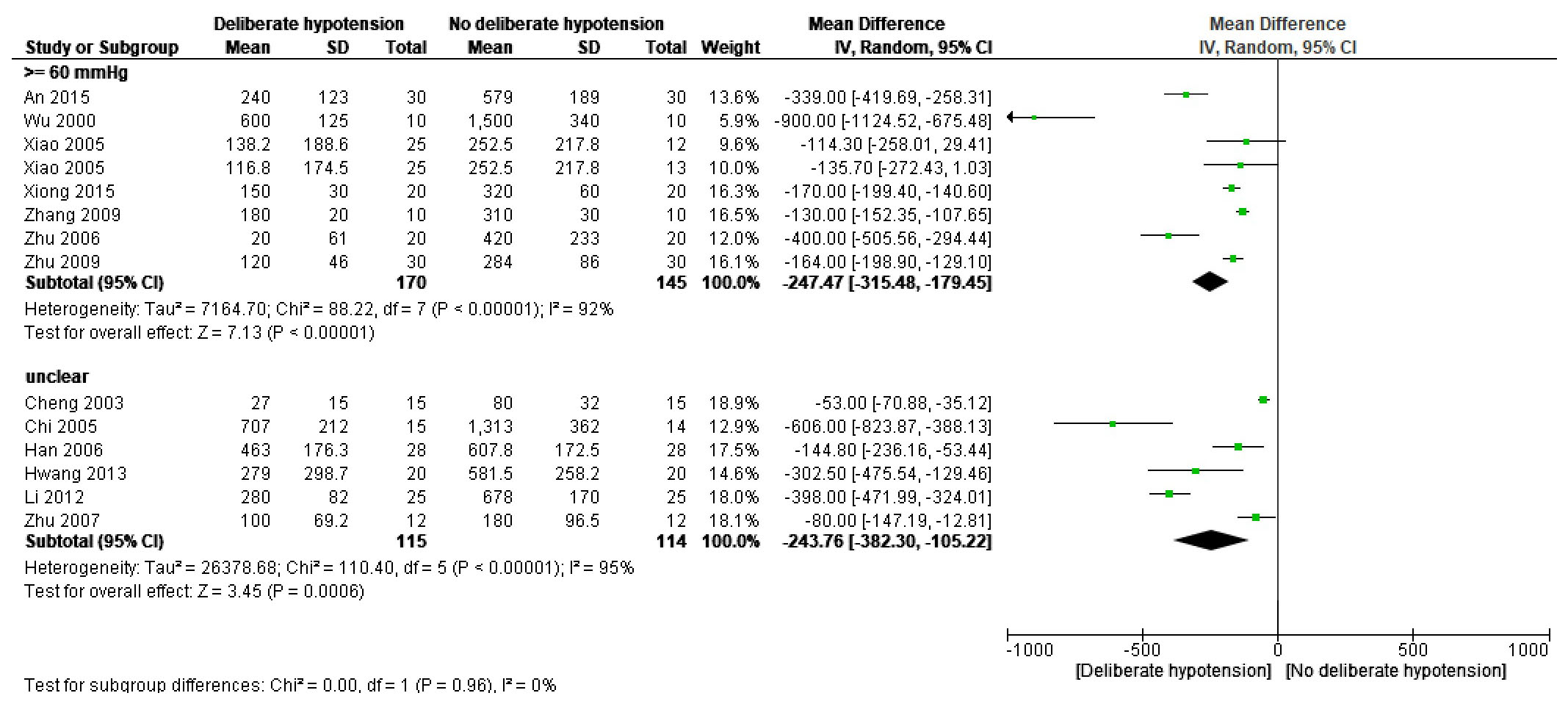


**Fig. S7** Forest plot for comparison of volume of blood transfused based on different controlled MAP levels between deliberate hypotension and no deliberate hypotension. IV, Inverse Variance.


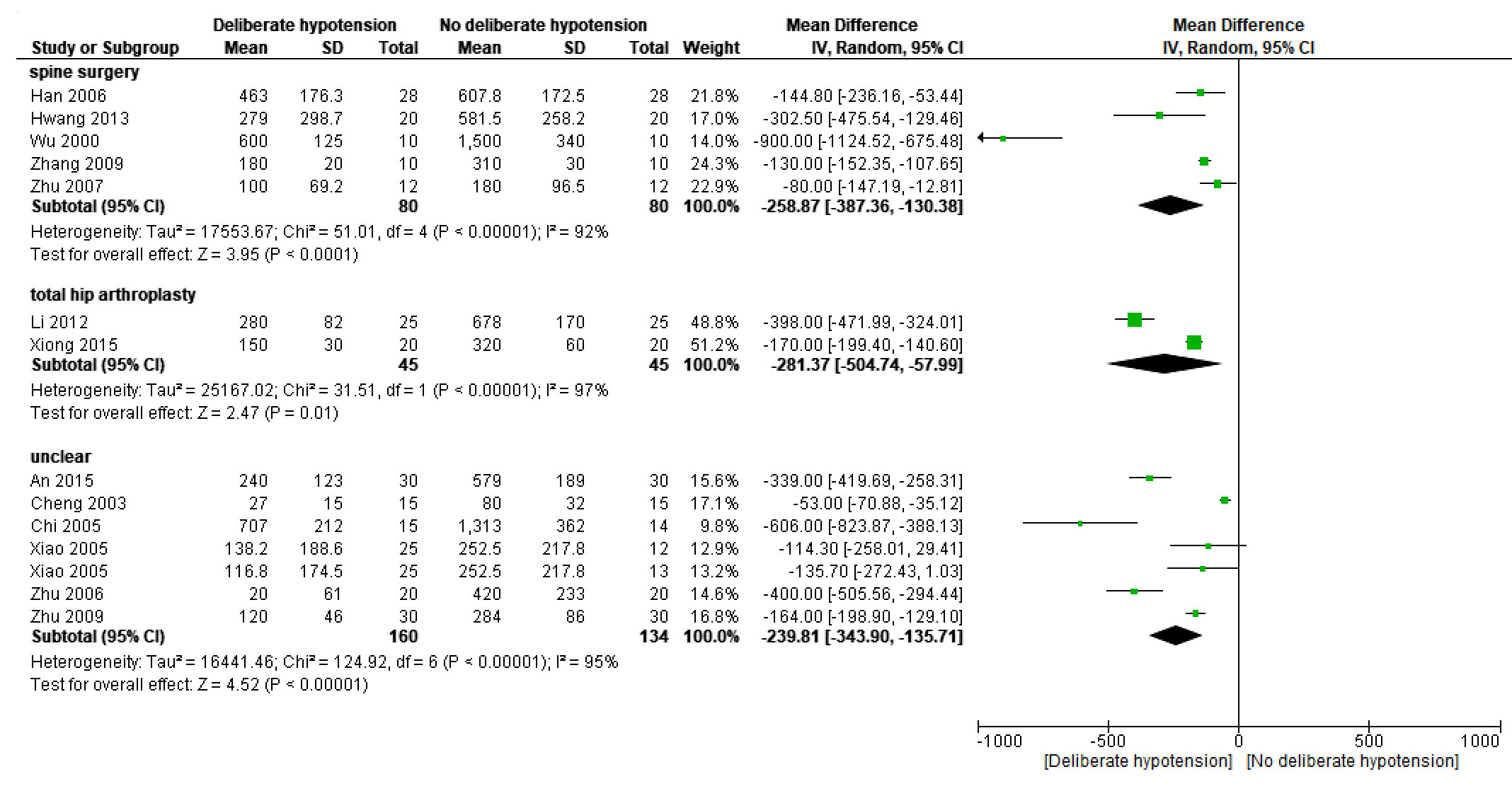


**Fig. S8** Forest plot for comparison of volume of blood transfused based on different types of orthopedic surgeries between deliberate hypotension and no deliberate hypotension. IV, Inverse Variance.


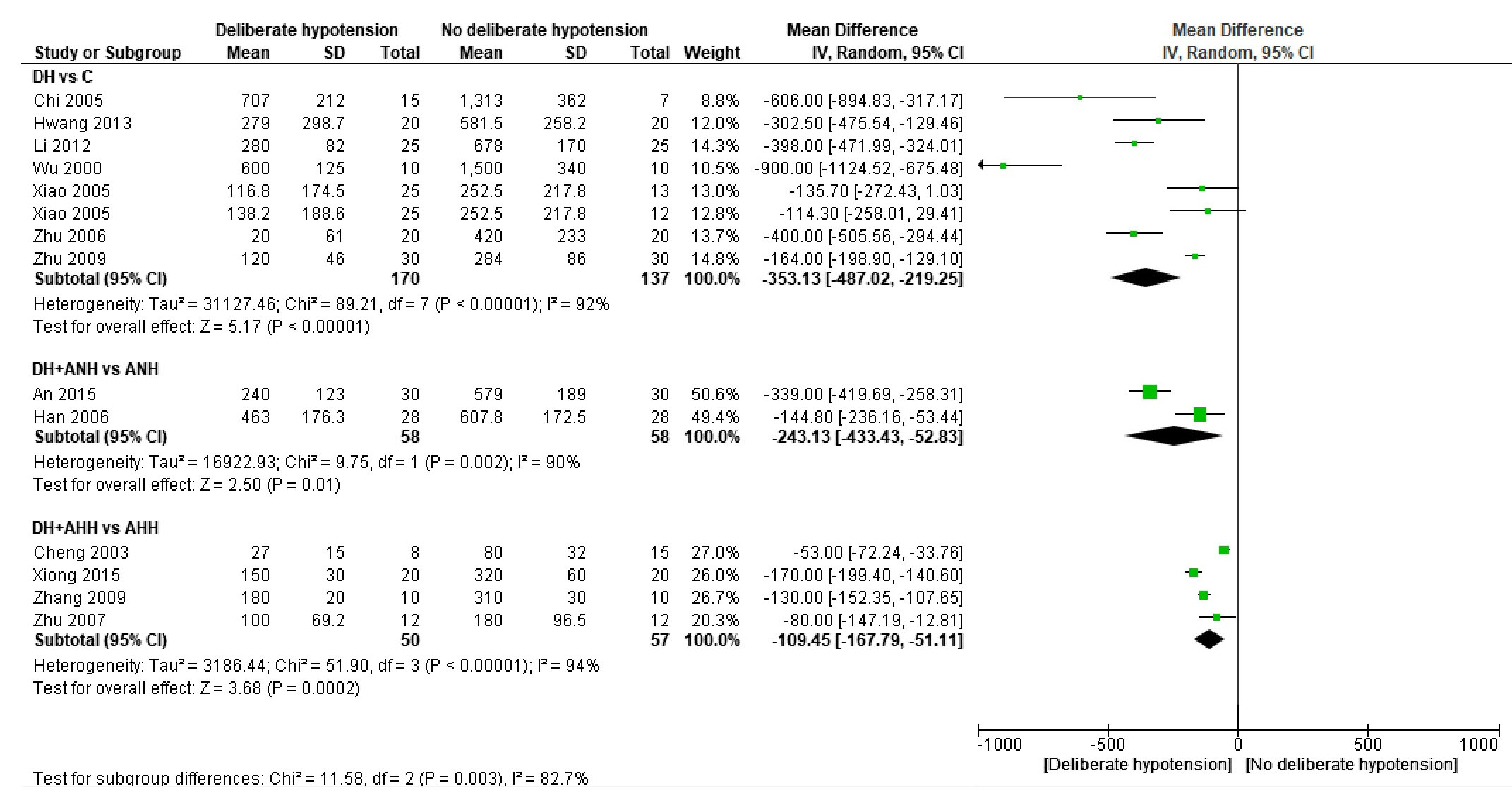


**Fig. S9** Forest plot for comparison of volume of blood transfused based on different combinations of other blood conservative method between deliberate hypotension and no deliberate hypotension. IV, Inverse Variance; DH: deliberate hypotension; C: control; ANH: acute normovolemic hemodilution; AHH: acute hypervolemic hemodilution; A: autologous blood transfusion with cell salvage.


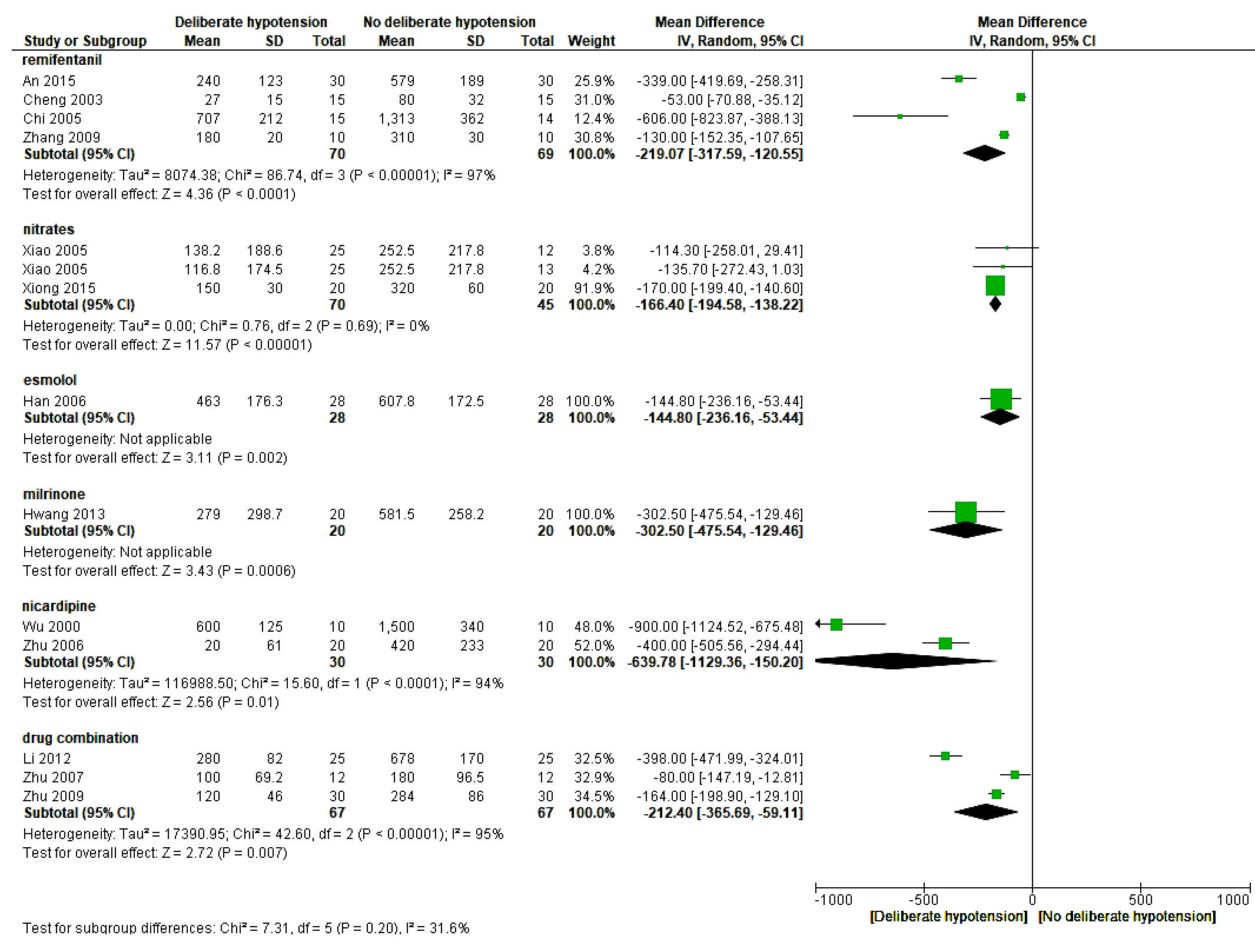


**Fig. S10** Forest plot for comparison of volume of blood transfused based on different hypotensive methods between deliberate hypotension and no deliberate hypotension. IV, Inverse Variance.
